# Ctdnep1 and Eps8L2 regulate dorsal actin cables for nuclear positioning during cell migration

**DOI:** 10.1016/j.cub.2021.01.007

**Published:** 2021-04-12

**Authors:** Francisco J. Calero-Cuenca, Daniel S. Osorio, Sofia Carvalho-Marques, Sreerama Chaitanya Sridhara, Luis M. Oliveira, Yue Jiao, Jheimmy Diaz, Cátia S. Janota, Bruno Cadot, Edgar R. Gomes

**Affiliations:** 1 Instituto de Medicina Molecular João Lobo Antunes, Faculdade de Medicina da Universidade de Lisboa, Avenida Professor Egas Moniz, 1649-028 Lisboa, Portugal; 2 Center for Research in Myology, INSERM U974, CNRS FRE3617, Université Pierre et Marie Curie, Sorbonne Universités, GH Pitié Salpêtrière, 75013 Paris, France; 3 Instituto de Histologia e Biologia do Desenvolvimento, Faculdade de Medicina, Universidade de Lisboa, Avenida Professor Egas Moniz, 1649-028 Lisboa, Portugal

**Keywords:** LINC Complex, actin, Ctdnep1, Eps8L2, nuclear positioning, TAN lines, cell migration

## Abstract

Cells actively position their nuclei within the cytoplasm for multiple cellular and physiological functions.^1^, ^2^, ^3^ Consequently, nuclear mispositioning is usually associated with cell dysfunction and disease, from muscular disorders to cancer metastasis.^4^, ^5^, ^6^, ^7^ Different cell types position their nuclei away from the leading edge during cell migration.^8^, ^9^, ^10^, ^11^ In migrating fibroblasts, nuclear positioning is driven by an actin retrograde flow originated at the leading edge that drives dorsal actin cables away from the leading edge. The dorsal actin cables connect to the nuclear envelope by the linker of nucleoskeleton and cytoskeleton (LINC) complex on transmembrane actin-associated nuclear (TAN) lines.^12^, ^13^, ^14^ Dorsal actin cables are required for the formation of TAN lines. How dorsal actin cables are organized to promote TAN lines formation is unknown. Here, we report a role for Ctdnep1/Dullard, a nuclear envelope phosphatase,^15^, ^16^, ^17^, ^18^, ^19^, ^20^, ^21^, ^22^ and the actin regulator Eps8L2^23^, ^24^, ^25^ on nuclear positioning and cell migration. We demonstrate that Ctdnep1 and Eps8L2 directly interact, and this interaction is important for nuclear positioning and cell migration. We also show that Ctdnep1 and Eps8L2 are involved in the formation and thickness of dorsal actin cables required for TAN lines engagement during nuclear movement. We propose that Ctdnep1-Eps8L2 interaction regulates dorsal actin cables for nuclear movement during cell migration.

## Highlights

• Ctdnep1 and Eps8L2 are required for nuclear positioning and TAN lines formation
• Ctdnep1 directly interacts with Eps8L2 for nuclear movement and cell migration
• Ctdnep1-Eps8L2 interaction regulates dorsal actin organization

## Graphical abstract

**Figure:**
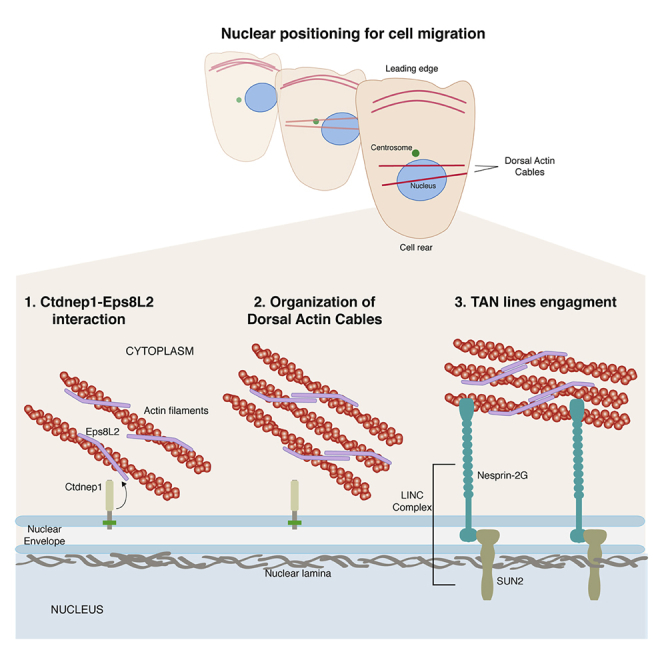

## Teaser

Nucleo-cytoskeletal connections are crucial for nuclear positioning. Calero-Cuenca et al. show that Ctdnep1 and Eps8L2 interact to regulate perinuclear actin cables organization promoting transmembrane actin-associated nuclear (TAN) lines formation. Ctdnep1-Eps8L2 interaction is required for nuclear positioning and proper cell migration.

## Results and discussion

### Ctdnep1 and Eps8L2 are required for nuclear positioning and cell migration in fibroblasts

The linker of nucleoskeleton and cytoskeleton (LINC) complex is the main player connecting the nucleus to the cytoskeleton, and it has an essential role in nuclear movement and positioning.^26^, ^27^, ^28^ To identify new regulators of nuclear position, we decided to study the involvement of Ctdnep1, a nuclear envelope transmembrane Ser/Thr phosphatase,^17^^,^^29^ in polarization of migrating cells. Ctdnep1 is involved in neuronal development (a complex process that requires tight regulation of nuclear positioning pathways)^1^^,^^30^ and nuclear membrane biogenesis.^17^^,^^18^ Interestingly, mutations in *CTDNEP1* that result in the loss of wild-type allele have been associated with medulloblastoma progression, the most common type of primary brain tumor in childhood.^31^^,^^32^ Additionally, because Ctdnep1 is a phosphatase,^17^ the possibility of regulation of nuclear movement by phosphorylation established an interesting hypothesis to investigate.

**Figure 1:**
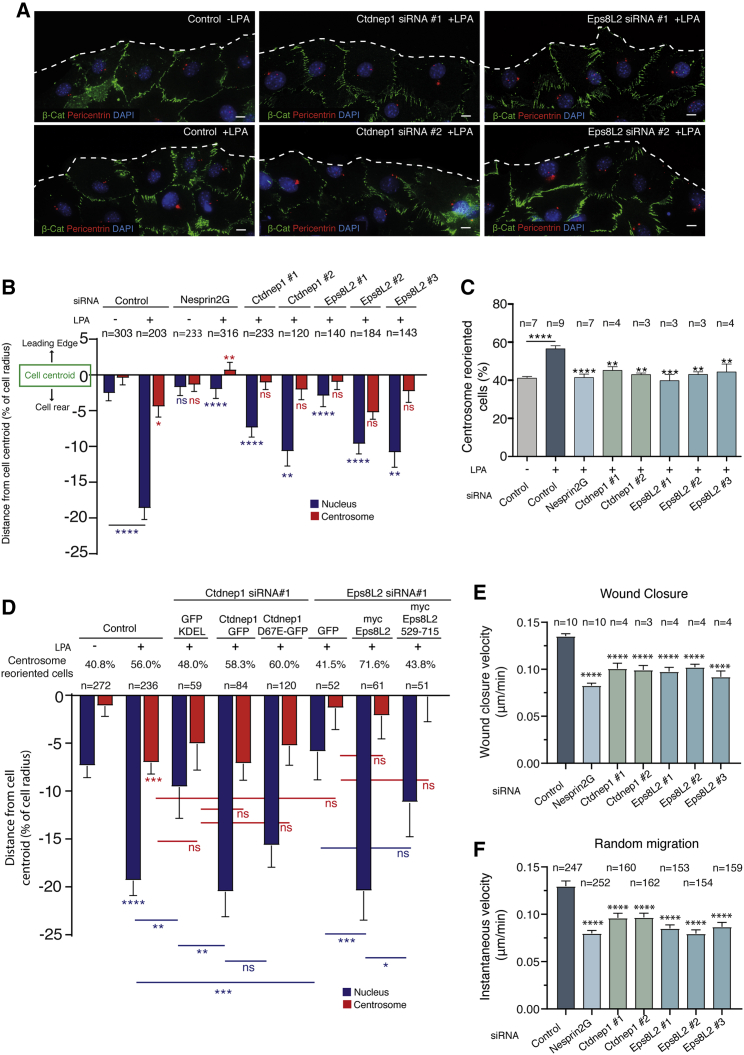
Ctdnep1 and Eps8L2 are required for nuclear positioning and cell migration in fibroblasts (A) Representative images of wound-edge 3T3 fibroblasts with or without LPA stimulation in control, Ctdnep1, and Eps8L2 siRNAs stained for β-catenin (green, cell contacts), pericentrin (red, centrosome), and DAPI (blue, nucleus). The dashed white lines mark the wound edge. (B) Average positions of the nucleus (blue) and centrosome (red) relative to the cell centroid in cells treated with control, Nesprin2G, Ctdnep1, and Eps8L2 siRNAs. (C) Percentage of oriented cells in the conditions analyzed in (B). (D) Average positions of the nucleus (blue) and centrosome (red) in cells treated with Ctdnep1 or Eps8L2 siRNAs and microinjected with KDEL-GFP, Ctdnep1-GFP, Ctdnep1_D67E-GFP (no phosphatase activity), GFP, myc-Eps8L2, or myc-Eps8L2_529-715. (E and F) Average wound closure velocity during wound closure migration (E) and instantaneous cell velocity in random migration (F) in cells treated with control, Nesprin2G, Ctdnep1, and Eps8L2 siRNAs. Data are represented as mean ± SEM. The n value means number of analyzed cells (B, D, and F) or number of experiments (C and E) with >50 cells analyzed per experiment. Statistics was performed by unpaired t test: ^∗^p < 0.05; ^∗∗^p < 0.01; ^∗∗∗^p < 0.001; ^∗∗∗∗^p < 0.0001. Scale bars 10μm. See also Figure S1.

To study the role of Ctdnep1 in nuclear positioning, we used small interference ribonucleic acid (siRNA) to deplete Ctdnep1 in NIH 3T3 fibroblasts (Figure S1A) grown to confluence and serum starved for 48 h. We wounded the monolayer and stimulated cell polarization by adding Oleyl-L-α-lysophosphatidic acid sodium salt (LPA) (Figure 1A), as previously described.^12^^,^^33^ We quantified nuclei and centrosomes positions relative to the cell centroid and centrosome reoriented cells (Figures 1B and 1C). Cells treated with control siRNA positioned their nuclei away from the cell centroid upon LPA stimulation, whereas the nucleus of non-LPA-treated cells remained near the cell centroid. When the LINC complex was disrupted by Nesprin2G depletion, nuclear positioning away from the cell centroid was inhibited (Figure 1B) and centrosome did not reorient (Figure 1C), as previously shown.^13^^,^^34^ Interestingly, upon depletion of Ctdnep1 using different siRNA oligos, nuclear positioning away from the cell centroid and centrosome reorientation were also inhibited (Figures 1A–1C), thus suggesting a role for Ctdnep1 on nuclear positioning.

We then questioned whether the phosphatase activity of Ctdnep1 was required for nuclear positioning. We microinjected siRNA-resistant Ctdnep1-GFP and Ctdnep1_D67E-GFP (a mutant with no phosphatase activity)^19^ as well as GFP-KDEL (KDEL is a target peptide to the ER and nuclear envelope) as a control in starved wound-edge fibroblasts transfected with Ctdnep1 siRNA. We observed that, upon LPA stimulation, Ctdnep1-GFP fully restored nuclear positioning away from the cell centroid and Ctdnep1_D67E-GFP partially rescued this nuclear positioning (Figure 1D). Therefore, the phosphatase activity of Ctdnep1 does not seem to be involved in nuclear movement.

To identify the molecular mechanism by which Ctdnep1 regulates nuclear positioning, we performed a yeast two-hybrid (Y2H) screen using the cytoplasmic domain of Ctdnep1 as bait to identify potential Ctdnep1 interacting partners. The only actin-binding protein identified was Eps8L2, a member of Eps8 family (Figure S1B). Eps8 family is characterized by a C-terminal actin-binding domain, with actin capping and bundling activity, and this protein family is involved in filopodia and stereocilia formation, as well as cell migration by Rac1 activation.^23^, ^24^, ^25^^,^^35^, ^36^, ^37^ In order to address the role of Eps8L2 in nuclear positioning, we depleted Eps8L2 from fibroblasts using different siRNA oligos (Figure S1A). Transient depletion of Esp8L2 reduced nuclear positioning away from the cell centroid (Figures 1A and 1B) as well as centrosome reorientation (Figure 1C), similarly to Ctdnep1-depleted fibroblasts. Furthermore, expression of Eps8L2 full length by microinjection in Eps8L2 siRNA cells rescued nuclear positioning, further supporting a role for Eps8L2 on nuclear positioning (Figure 1D).

Nuclear positioning and centrosome reorientation are important for cell migration.^9^^,^^38^ Therefore, we investigated the role of Ctdnep1 and Eps8L2 during cell migration using wound closure and random migration assays. Fibroblasts depleted for Ctdnep1 and Eps8L2 migrated less when compared to control cells in both wound closure and random migration assays (Figures 1E, 1F, and S1C). Additionally, cell directionality and migration persistence were also affected in cells at the wound edge during wound closure migration (Figures S1D–S1F). All together, these results indicate that Ctdnep1 and Eps8L2 are required for nuclear positioning, centrosome reorientation, and proper cell migration in fibroblasts.

### Ctdnep1 interacts directly with Eps8L2 independently of Ctdnep1 phosphatase activity

Taking into account that Ctdnep1 and Eps8L2 showed a positive interaction in the Y2H assay and are both required for nuclear positioning, we analyzed the subcellular localization of Ctdnep1 and Eps8L2 in migrating fibroblasts. Due to the lack of suitable antibodies for immunohistochemistry, we performed microinjection of cDNA encoding Ctdnep1, Ctdnep1_D67E, and Eps8L2 in wound-edge cells. Ctdnep1 localizes to the nuclear envelope and endoplasmic reticulum (ER), as we observed by its colocalization with the ER protein Sec61β (Figure S1G).^39^ Using digitonin permeabilization, we also noted that Ctdnep1 is at the outer nuclear membrane (Figure S1H). On the other hand, Eps8L2 localizes in the cytoplasm and is enriched at the leading edge, actin filaments, cell projections, and perinuclear region (Figure S2A). We quantified the colocalization of Ctdnep1 and Eps8L2 and found an increase in the perinuclear area (Figure S2B).

**Figure 2:**
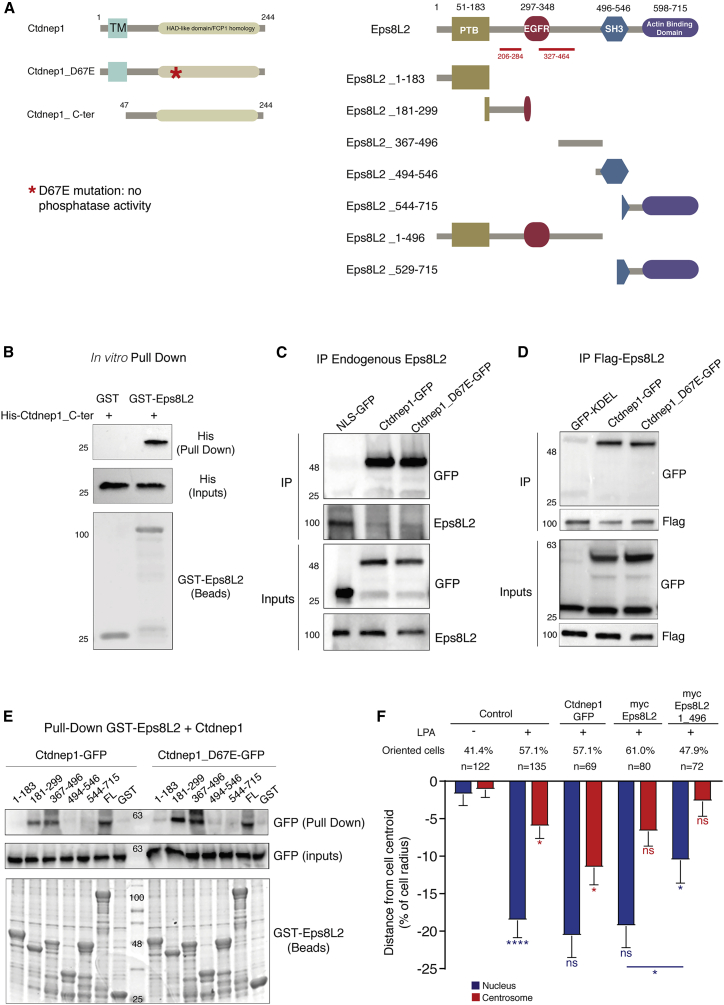
Ctdnep1 and Eps8L2 directly interact (A) Schematic representation of Ctdnep1, Ctdnep1_D67E (red asterisk denotes D67E mutation), Ctdnep1_C-ter (Ctdnep1 without the transmembrane domain), and Eps8L2 proteins showing their different protein domains and fragments used in this work. TM indicates transmembrane domain. Red lines in Eps8L2 indicate the regions of interaction with Ctdnep1 obtained in the Y2H assay. (B) *In vitro* pull-down of recombinant GST-Eps8L2 bound to glutathione agarose beads with purified recombinant His-Ctdnep1_C-ter. (C) Co-immunoprecipitation of endogenous Eps8L2 from SRKB cells with NLS-GFP, Ctdnep1-GFP, and Ctdnpe1_D67E-GFP from transfected U2OS cells. (D) Co-immunoprecipitation of FLAG-Eps8L2 overexpressed in U2OS cells and posteriorly incubated with GFP-KDEL, Ctdnep1-GFP, and Ctdnep1_D67E-GFP overexpressed in U2OS cells. (E) Pull-down assay of recombinant GST-Eps8L2 and its different fragments bound to glutathione agarose beads with Ctdnep1-GFP and Ctdnep1_D67E-GFP overexpressed in U2OS cells. (F) Average positions of the nucleus (blue) and centrosome (red) in wild-type cells microinjected with Ctdnep1-GFP, myc-Eps8L2, or myc-Eps8L2_1-496. The n value means number of analyzed cells. Data are represented as mean ± SEM. Statistics was performed by unpaired t test: ^∗^p < 0.05; ^∗∗^p < 0.01; ^∗∗∗^p < 0.001; ^∗∗∗∗^p < 0.0001. See also Figure S2.

We then investigated whether Ctdnep1 physically interacts with Eps8L2 and whether this interaction was direct. We performed an *in vitro* pull-down using recombinant His-Ctdnep1_Cter, a cytoplasmic version of Ctdnep1 without the transmembrane domain (to make the protein soluble; Figure 2A). We found His-Ctdnep1_Cter strongly pulled down glutathione S-transferase (GST)-Eps8L2, suggesting that both proteins interact directly (Figure 2B). Additionally, we investigated whether the Ctdnep1 phosphatase activity was involved in this interaction. We immunoprecipitated endogenous Eps8L2 or FLAG-Eps8L2 co-expressed with Ctdnep1-GFP or Ctdnep1_D67E-GFP. We were able to co-immunoprecipitate endogenous and expressed Eps8L2 with both Ctdnep1 constructs (Figures 2C and 2D), suggesting that the phosphatase activity of Ctdnep1 is not required for the interaction. Overall, these results indicate that the Ctdnep1-Eps8L2 physical interaction is direct and the phosphatase activity of Ctdnep1 is not necessary for its interaction with Eps8L2.

It was previously reported that phosphorylation of Eps8 modulates its function during axonal filopodia formation.^36^ For Eps8L2, different reports identified several amino acids that can be phosphorylated.^40^, ^41^, ^42^ Therefore, we tested whether Eps8L2 was a Ctdnep1 substrate. We performed Phos-tag SDS-PAGE to analyzed Eps8L2 phosphorylation in SKBR3 cell lysates (which have high levels of endogenous Eps8L2)^43^ treated with control and Ctdnep1 siRNAs. As positive control, we added Lambda phosphatase to the lysates to dephosphorylate all proteins in the sample. We found that Eps8L2 band appeared in a lower molecular weight in Lambda-treated lysates, suggesting that Eps8L2 is phosphorylated as previously described (Figure S2C). However, we did not observe any differences between control or Ctdnep1 siRNAs (Figure S2C). Additionally, we performed mass spectrometry to identify phosphorylation sites in myc-Eps8L2 purified from U2OS cells co-transfected with Ctdnep1-GFP or Ctdnep1_D67E-GFP. We identified several phosphorylated sites in Eps8L2, some previously reported. However, we did not observe any difference regarding the phosphorylation profile related to the phosphatase activity of Ctdnep1 when we compared the different conditions (Figure S2D).

### Ctdnep1-Eps8L2 interaction regulates nuclear positioning in migrating fibroblasts

Eps8L2 contains four described domains: PTB; EGFR; SH3; and an actin-binding domain at the C-terminal (Figure 2A).^25^^,^^44^ We cloned different fragments of Eps8L2 and produced recombinant protein of each fragment tagged with GST (Figure 2A). We then performed pull-down assays using extracts of U2OS cells expressing Ctdnep1-GFP or Ctdnep1_D67E-GFP. We observed that Ctdnep1-GFP and Ctdnep1_D67E-GFP were pulled down by Eps8L2 fragments 181–299 and 367–496, to a similar extent as full-length Eps8L2 (Figure 2E). These regions overlap with the two regions detected in our Y2H screen, thus strongly suggesting that the N-terminal region between the PBT and SH3 domain of Eps8L2 interacts with Ctdnep1.

To test the role of the interaction between Ctdnep1 and Eps8L2 on nuclear position, we used an N-terminal Eps8L2 construct (Eps8L2_1-496) that includes both regions known to interact with Ctdnep1 (Figure S2E). Eps8L2_1-496 can thus disrupt Ctdnep1-Eps8L2 interaction by a dominant negative effect. We found that expression of Eps8L2_1-496, but not Ctdnep1 or Eps8L2 full length, impaired nuclear positioning (Figure 2F). Furthermore, we performed rescue experiments in Eps8L2 siRNA cells using Eps8L2 fragment that does not interact with Ctdnep1 (Eps8L2_529-715; Figure S2E) and did not observe restoration of nuclear position (Figure 1D). Finally, we tested whether the identified interaction is involved in cell migration by microinjecting dominant-negative Eps8L2_1-496 construct in leading edge fibroblasts and measured their persistence at the leading edge during wound closure. We found a reduction of wound-edge cells expressing Eps8L2_1-496 relative to cells expressing Eps8L2 full length (FL) (Figures S2F and S2G). Concomitantly, the distance between microinjected cells and wound edge was higher for Eps8L2_1-496-expressing cells, when compared to Eps8L2 FL (Figure S2H). Overall, these results indicate that the interaction between Ctdnep1 and Eps8L2 is involved in nuclear positioning and cell migration.

### Ctdnep1 and Eps8L2 are required for TAN lines formation

Nuclear movement during centrosome reorientation is driven by actin retrograde flow and requires the formation of transmembrane actin-associated nuclear (TAN) lines by the connection of dorsal actin cables to the LINC complex.^12^^,^^13^ We first measured actin retrograde flow in fibroblasts stably expressing Lifeact-mCherry upon depletion of Ctdnep1 and Eps8L2. We observed that the speed of the actin retrograde flow near the leading edge and on the top of the nucleus, where the TAN lines are formed, was slightly increased upon Eps8L2 depletion whereas was not altered upon Ctdnep1 depletion (Figures S3A and S3B). Additionally, the percentage of cells with actin retrograde flow was slightly decreased in Ctdnep1- and Eps8L2-depleted cells when compared to control (Figure S3C). Therefore, the minor changes we observed on actin retrograde flow upon Ctdnep1 and Eps8L2 depletions cannot account for the observed effect on nuclear movement.

**Figure 3:**
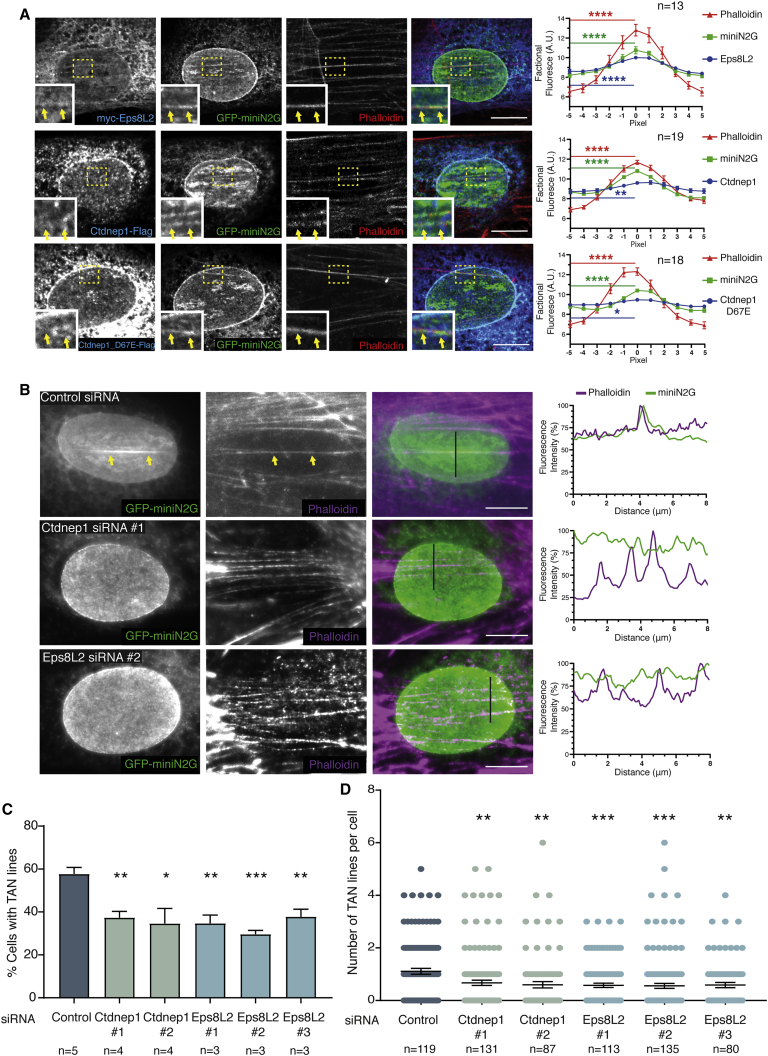
Ctdnep1 and Eps8L2 are required for TAN lines formation (A) Wound-edge fibroblast stimulated with LPA and microinjected with GFP-miniN2G and myc-Eps8L2 (top panels), Ctdnep1-FLAG (middle panels), and Ctdnep1_D67E-FLAG (bottom panels). Cells were stained for GFP (green), phalloidin (red), myc (blue), and FLAG (blue). Dashed yellow squares indicate the regions of the insets shown in the bottom left corner. Colocalization of miniN2G and actin (phalloidin) in linear arrays at the nuclear envelope is indicated by yellow arrows in the insets. Quantification of the colocalization between miniNesprin2G, phalloidin, and Eps8L2 or Ctdnep1 is shown in the right plots. For the quantification, an 11-pixels-size box (width) was designed with the TAN line centered in the central pixel in order to measure the intensity for each channel and for each row of pixels (see STAR methods). The n value means number of TAN lines analyzed. (B) Wound-edge fibroblasts transfected with control, Ctdnep1, and Eps8L2 siRNAs were microinjected with GFP-miniN2G to analyze TAN lines formation. Cells were stained for GFP (green) and phalloidin (magenta). TAN lines can be visualized in the control (yellow arrows). Line scans for each channel are represented in the right plots. Scale bars 10 μm. (C) Quantification of the percentage of cells presenting at least one TAN line in wound-edge fibroblasts treated with control, Ctdnep1, and Eps8L2 siRNAs. The n value means number of experiments with >25 cells per experiment. (D) Quantification of number of TAN lines per cell in the conditions described in (C). The n value means number of analyzed cells. Data are represented as mean ± SEM. Statistics was performed by unpaired t test: ^∗^p < 0.05; ^∗∗^p < 0.01; ^∗∗∗^p < 0.001; ^∗∗∗∗^p < 0.0001. See also Figure S3.

We then explored the role of Ctdnep1 and Eps8L2 in TAN lines dynamics. First, we evaluated whether Ctdnep1 or Eps8L2 were enriched at TAN lines. To address this question, we microinjected GFP-miniNesprin2G (miniN2G), a functional reporter of TAN lines,^13^ together with myc-Eps8L2, Ctdnep1-FLAG, or Ctdnep1_D67E-FLAG in wild-type wound-edge fibroblasts. Upon LPA stimulation, we observed that Eps8L2 colocalized with dorsal actin cables at TAN lines (Figure 3A). Although Ctdnep1 and Ctdnep1_D67E colocalization at TAN lines is not as striking as Eps8L2, quantification of Ctdnep1 and Ctdnep1_D67E at the nuclear envelope suggests a significant increase of Ctdnep1 at TAN lines (Figure 3A). We then tested whether Ctdnep1 or Eps8L2 were required for TAN lines formation. We microinjected GFP-miniN2G in wound-edge fibroblasts transfected with control, Ctdnep1, or Eps8L2 siRNAs, followed by LPA stimulation. Depletion of Ctdnep1 or Eps8L2 reduced the number of cells with TAN lines (Figures 3B and 3C). In addition, the average number of TAN lines per cell was decreased in cells depleted for Ctdnep1 or Eps8L2 (Figure 3D). These results suggest that Ctdnep1 and Eps8L2 are involved in TAN lines formation during nuclear positioning in migrating fibroblasts.

### Ctdnep1 and Eps8L2 regulate dorsal actin organization

**Figure 4:**
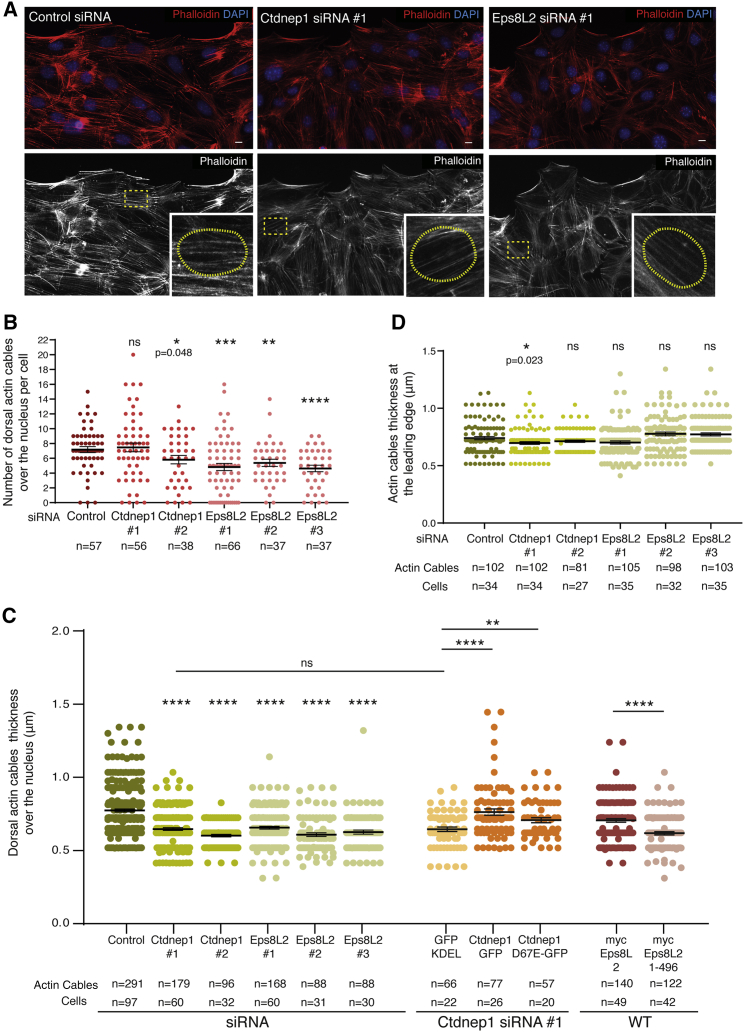
Ctdnep1 and Eps8L2 regulate dorsal actin cables organization (A) Representative images of wound-edge fibroblasts stimulated with LPA and treated with control, Ctdnep1, and Eps8L2 siRNAs and stained for phalloidin (red, actin) and DAPI (blue, nucleus). Dashed yellow squares indicate the regions of the insets shown in the bottom right corner. The yellow dot circles in the insets denote the nucleus border. Scale bars 10 μm. (B) Quantification of number of dorsal actin cables on top of the nucleus in cells treated with control, Ctdnep1, or Eps8L2 siRNAs. The n value means number of analyzed cells. (C) Quantification of dorsal actin cables thickness in cells treated with control, Ctdnep1, or Eps8L2 siRNAs in fibroblasts transfected with Ctdnep1 siRNA and microinjected with KDEL-GFP, Ctdnep1-GFP, or Ctdnep1_D67E-GFP and in wild-type cells microinjected with myc-Eps8L2 or myc-Eps8L2_1-496. (D) Quantification of actin cables thickness at the leading edge in cells treated with control, Ctdnep1, or Eps8L2 siRNAs. Statistics was performed by unpaired t test: ^∗^p < 0.05; ^∗∗^p < 0.01; ^∗∗∗^p < 0.001, ^∗∗∗∗^p < 0.0001. See also Figure S4.

TAN line formation requires actin retrograde flow and dorsal actin cables.^14^ Furthermore, we showed that Eps8L2 localizes to dorsal actin cables on TAN lines (Figure 3A) and both Ctdnep1 and Eps8L2 are required for TAN lines formation (Figures 3B–3D). Therefore, we investigated whether Ctdnep1 and Eps8L2 were specifically involved in the formation of dorsal actin cables. We examined dorsal actin organization over the nucleus in serum-starved wounded monolayer of fibroblasts treated with control, Ctdnep1, and Eps8L2 siRNAs after stimulation with LPA. We observed a decrease in the number of dorsal actin cables in cells depleted for Eps8L2 (Figures 4A and 4B), without any changes on focal adhesions (Figures S4A–S4C). We then analyzed in more detail the dorsal actin cables over the nucleus in the absence of Ctdnep1 and Eps8L2 and found a reduction of dorsal actin cables thickness when compared to control siRNA (Figure 4C). Interestingly, when we measured actin filament thickness in other regions of the cell, such as the leading edge (where the actin retrograde flow is originated), we did not observe differences between Ctdnep1 or Eps8L2 siRNAs and control (Figure 4D), suggesting a local effect of Ctdnep1 and Eps8L2 in actin organization. We then tested whether the phosphatase domain of Ctdnep1 was involved in regulating dorsal actin cables thickness. We microinjected siRNA-resistant Ctdnep1-GFP and Ctdnep1_D67E-GFP as well as GFP-KDEL as a control in starved wound-edge fibroblasts transfected with Ctdnep1 siRNA. We observed that Ctdnep1-GFP and Ctdnep1_D67E-GFP fully restored dorsal actin cables thickness (Figures 4C and S4D). Interestingly, we also observed that microinjection of dominant-negative Eps8L2 (Eps8L2_1-496), but not full-length Eps8L2, in wild-type fibroblasts decreases dorsal actin cables thickness (Figure 4C). These results support a role for Ctdnep1 and Eps8L2 at the nuclear periphery on the formation and maintenance of dorsal actin cables required for TAN lines formation, nuclear movement, and cell migration.

Nucleus-cytoskeleton connections are crucial for nuclear dynamics and cell function. Here, we showed that the nuclear envelope phosphatase Ctdnep1 and the actin-binding protein Eps8L2 are involved in the formation and maintenance of dorsal actin filaments required for the engagement of the LINC complex at TAN lines for nuclear movement.

We demonstrated that Ctdnep1 and Eps8L2 directly interact. Additionally, we provided evidence for a role of this interaction on nuclear movement, in a dephosphorylation-independent manner. The bundling activity of the actin-binding domain of Eps8 has been shown previously.^24^ This domain shares high similarity within Eps8 family members. Therefore, Eps8L2 might also have bundling activity.^24^^,^^25^ Ctdnep1-Eps8L2 interaction may be transient but essential to regulate Eps8L2 bundling activity stabilizing actin filaments that can properly organize LINC complexes into TAN lines for nuclear movement.

Ctdnep1 depletion does not affect the overall actin organization in migrating fibroblasts. Conversely, when we reduced Eps8L2 protein levels, we observed a reduction of dorsal actin cables on the top of the nucleus without affecting focal adhesions and stress fibers. Both Ctdnep1 and Eps8L2 depletions lead to a decrease in dorsal actin cables thickness, probably due to the lack of a possible Eps8L2 bundling activity. Thinner dorsal actin cables would impair TAN lines formation due to (1) a faster speed in actin cables retrograde flow and (2) reduced contact surface or traction forces between thinner actin cables and LINC complex. Both scenarios would reduce the ability of actin cables to bind to the LINC complex and to engage TAN lines for nuclear movement.

Ctdnep1 function on lipid metabolism and nuclear membrane biogenesis depends on Ctdnep1 phosphatase activity.^15^^,^^45^ We did not find any role for the phosphatase activity of Ctdnep1 on nuclear movement, interaction with Eps8L2, or dorsal actin cables regulation. Furthermore, we did not detect any changes in the phosphorylation of Eps8L2 dependent on the phosphatase activity of Ctdnep1. Although our results do not entirely discard Eps8L2 dephosphorylation by Ctdnep1, they strongly suggest a dephosphorylation-independent role of Ctdnep1 and Eps8L2 on the regulation of dorsal actin cables for nuclear positioning. The mechanism by which Ctdnep1 is controlling Eps8L2 activity is still elusive, as well as how Ctdnep1-Eps8L2 interaction and regulation of actin organization are restricted to the perinuclear region. Further studies are necessary to shed light on these questions.

In the last years, perinuclear actin organization and function have been revealed important for nuclear positioning and cell migration.^46^^,^^47^ Additionally, the LINC complex has a central role for mechanosensing and mechanotransduction, due to its function as a bridge between the cytoskeleton and the nucleus.^48^, ^49^, ^50^, ^51^ Nevertheless, how this LINC complex function is regulated as well as the different cellular processes where it is involved needs to be deciphered. The role of Ctdnep1-Eps8L2 interaction on actin organization described in this report can therefore be involved in the multiple functions of LINC complex-mediated nucleus-cytoskeleton connection.

## STAR★Methods

### Key resources table

**Table:** 

REAGENT or RESOURCE	SOURCE	IDENTIFIER
**Antibodies and probes**		

Rabbit anti-β-Catenin	Invitrogen	#71-2700; RRID:AB_2533982
Mouse anti-Pericentrin	BD-Biosciences	#611814; RRID:AB_399294
Mouse anti-Flag	Sigma-Aldrich	#F1804; RRID:AB_262044
Chicken anti-GFP	Aves Lab	#GFP-1020; RRID:AB_10000240
Rabbit anti-LaminB1	Abcam	#ab16048; RRID:AB_443298
Mouse anti-Vinculin	Sigma-Aldrich	V9131; RRID:AB_477629
Mouse anti-Myc	Life Technologies	#13-2500; RRID:AB_2533008
Mouse anti-6x His	Abcam	#ab18184; RRID:AB_444306
Goat anti-Chicken IgY (H+L) Secondary Antibody, Alexa Fluor® 488 conjugate	ThermoFisher Scientific	#A-11039; RRID:AB_2534096
Donkey Anti-Mouse IgG (H+L) Highly Cross-Adsorbed Secondary Antibody, Alexa Fluor® 488	ThermoFisher Scientific	#A21202; RRID:AB_141607)
Donkey Anti-Rabbit IgG (H+L) Highly Cross-Adsorbed Secondary Antibody, Alexa Fluor® 488	ThermoFisher Scientific	#A21206; RRID:AB_2535792
Goat anti-Mouse IgG (H+L) Secondary Antibody, Alexa Fluor® 555 conjugate	ThermoFisher Scientific	#A21424; RRID:AB_141780
Goat Anti-Rabbit IgG (H+L) Highly Cross-Adsorbed Secondary Antibody, Alexa Fluor® 555	ThermoFisher Scientific	#A21429; RRID:AB_2535850
Goat anti-Rabbit IgG (H+L) Secondary Antibody, Alexa Fluor® 647 conjugate	ThermoFisher Scientific	#A-21245; RRID:AB_2535813
Goat anti-Mouse IgG (H+L) Cross-Adsorbed Secondary Antibody, Alexa Fluor® 647	ThermoFisher Scientific	#A-21236; RRID:AB_2535805
Alexa Fluor® 488 Phalloidin	Life Technologies	#A12379
Alexa Fluor® 647 Phalloidin	Life Technologies	#A22287
DAPI	Sigma-Aldrich	#32670-5MG-F
Janelia Fluor® 646 HaloTag®	Promega	GA1120

**Bacterial and virus strains**		

Rosetta™ 2(DE3)pLysS	MERCK	71403-4
One Shot® TOP10 Chemically Competent *E. coli*	Invitrogen	C404010

**Biological samples**		

Bovine Calf Serum	Thermo Fisher Scientific	35-053-CM
Fetal Bovine Serum	Eurobio	CVFSVF00-01

**Chemicals, peptides, and recombinant proteins**		

LPA	Sigma-Aldrich	L7260
DpnI endonuclease	Thermo Fisher Scientific	ER1701
DNaseI recombinant	Roche	04716728001
High capacity RNA-to-cDNA kit	Thermo Fisher Scientific	4388950
TRIzol™ Reagent	Thermo Fisher Scientific	15596026
Power SYBR Green PCR MasterMix	Thermo Fisher Scientific	4368577
Lipofectamine 300	Invitrogen	L3000001
Lipofectamine RNAiMAX Transfection Reagent	Invitrogen	13778100
MEM Non-essential amino acids solution (100X)	Thermo Fisher Scientific	11140050
Penicillin/streptomycin (10000 U/ml)	Thermo Fisher Scientific	15140122
HEPES 1M	Thermo Fisher Scientific	15630106
Puromycin	Sigma-Aldrich	P9620-10ML
Mitomycin C	Santa Cruz Biotechnology	sc-3514
Digitonin	Sigma-Aldrich	D141-100MG
Triton X-100	Sigma-Aldrich	X100
N-Lauroylsarcosine sodium salt solution (Sarkosyl)	Sigam-Aldrich	61747-100ML
BlueSafe	NZYTech	MB15201
Pefabloc®	Roche	11585916001
IPTG	Zymo Research	I1001-25
SDS Sample Buffer	MERCK	70607
Glutathione Sepharose 4B Beads	GE Healthcare	17-0756-01
Pierce™ Protein A/G Magnetic Beads	Thermo Fisher Scientific	88802
Myc-Trap®_A beads	Chromotek	yta-20
Binding control agarose beads	Chromotek	bab-20
Fluoromount-G	Thermo Fisher Scientific	00-4958-02

**Experimental models: cell lines**		

U2OS cells	ATCC	HTB-96™
SKRB-3 cells	ATCC	HTB-30
HEK293T cells	ATCC	CRL-3216
NIH 3T3 cells	ATCC	CRL-1658

**Oligonucleotides**		

Primers for plasmids construction	This work	See Table S1
Primers for RT-qPCR	This work	See Table S1
siRNAs	This work	See Table S1

**Recombinant DNA**		

pEZYflag	Addgene	#18700
pDEST15	Fanny Jaulin Lab	N/A
pRK5mycGW	Fanny Jaulin Lab	N/A
pDONR201-Ctdnep1	This work	N/A
pDONR201-Ctdnep1_D67E	This work	N/A
pDONR201-Ctdnep1_C-ter	This work	N/A
pDEST47-Ctdnep1_WT	This work	N/A
pDEST47-Ctdnep1_D67E	This work	N/A
pDEST17-Ctdnep1_C-ter	This work	N/A
pDONR201-Eps8L2	This work	N/A
pDONR201-Eps8L2_1-183	This work	N/A
pDONR201-Eps8L2_181-299	This work	N/A
pDONR201-Eps8L2_297-370	This work	N/A
pDONR201-Eps8L2_367-496	This work	N/A
pDONR201-Eps8L2_494-546	This work	N/A
pDONR201-Eps8L2_544-715	This work	N/A
pDONR201-Eps8L2_529-715	This work	N/A
pDEST15- Eps8L2	This work	N/A
pDEST15- Eps8L2_1-183	This work	N/A
pDEST15- Eps8L2_181-299	This work	N/A
pDEST15- Eps8L2_297-370	This work	N/A
pDEST15- Eps8L2_367-496	This work	N/A
pDEST15- Eps8L2_494-546	This work	N/A
pDEST15- Eps8L2_544-715	This work	N/A
pDEST15-Eps8L2_529-715	This work	N/A
pGEX-6P-1-Eps8L2_1-496	This work	N/A
pRK5mycGW- Eps8L2	This work	N/A
pRK5mycGW-Eps8L2_529-715	This work	N/A
pEZYflag-Eps8L2	This work	N/A
pcDNA3.1+C-MYC-Eps8L2	This work	N/A
pcDNA3.1+C-MYC-Eps8L2_1-496	This work	N/A
pcDNA3.1(+)-C-eGFP- Ctdnep1_WT_siR	This work	N/A
pcDNA3.1(+)-C-eGFP- Ctdnep1_D67E_siR	This work	N/A
pDONR201	Thermo Fisher Scientific	N/A
pDEST47	Thermo Fisher Scientific	12281010
NLS-GFP	Jan Lammerding Lab	N/A
pUBC-GFP-KDel	[52]	N/A
pDEST17	Fanny Jaulin Lab	N/A
pLALI_Lifeact-mcherry	Olivier Pertz Lab	N/A
HaloTag-Sec61β	Jennifer Lippincott-Schwartz Lab	N/A
pEGFP-C1-GFP-miniNesprin2G	^13^	N/A
pcDNA3.1-Ctdnep1-2xFlag	^16^	N/A
pcDNA3.1-Ctdnep1_D67E-2xFlag	This work	N/A
pGEX-6P-1	MERCK	GE28-9546-48
pCMV-dR8.91	Olivier Pertz Lab	N/A
pCMV-VSV-G	Olivier Pertz Lab	N/A

**Software and algorithms**		

Cell Plot	^33^	https://changlab.fhs.um.edu.mo/software/
Fiji	ImageJ	https://imagej.net/Fiji
Adobe Photoshop CS5	Adobe	https://www.adobe.com
Adobe Illustrator CS5	Adobe	https://www.adobe.com
Zen	Zeiss	https://www.zeiss.com/microscopy/int/products/microscope-software/zen.html
Chemotaxis and Migration Tool	Ibidi	https://ibidi.com/chemotaxis-analysis/171-chemotaxis-and-migration-tool.html

**Other**		

SuperSep™Phos-tag™ 7.5%	Wako Chemicals	198-17987
Mini-PROTEAN® TGX 4-15%	BioRad	4561084

### Resource availability

#### Lead contact

Further information and requests for resources and reagents should be directed to and will be fulfilled by the Lead Contact, Edgar R Gomes (edgargomes@medicina.ulisboa.pt)

#### Materials availability

The plasmids generated in this study are available on request. Other reagents can also be made available on request.

#### Data and code availability

This study did not generate unique code. Dataset from the Yeast-2-Hybrid assay is shown in the manuscript.

### Experimental model and subject details

#### Cell lines

Low-passage NIH 3T3 fibroblasts (ATCC) were cultured in DMEM (Thermo Fisher Scientific) with no sodium pyruvate, 10% bovine calf serum, 10 mM HEPES and penicillin/streptomycin at 500 units/ml. U2OS cells (ATCC) were cultured in DMEM with sodium pyruvate, 10% Fetal Bovine Serum, and penicillin/streptomycin at 500 units/ml. SKRB-3 cells (ATCC) were cultured in DMEM with sodium pyruvate, 10% Fetal Bovine Serum, 1% Non-essential amino acids and penicillin/streptomycin at 500 units/ml. All cells lines were cultured in 5% CO_2_ at 37°C. All cells lines used in this work were obtained from the ATCC repository.

### Method details

#### Plasmids

Ctdnep1 was directly cloned from an NIH 3T3 mRNA library using the SuperScript® III One-Step RTPCR System (Life Technologies) using primers Ctdnep1_FL_N1_For and Ctdnep1_FL_N1_Rev that include AttB recombination site to pDONR201 Gateway donor vectors (Life Technologies). Ctdnep1_C-ter was amplified from Ctdnep1 Full Length (FL) entry vector by using primers Ctdnep1_Cter_C1_For and Ctdnpe1_FL_C1_Rev. For phosphatase null point mutant Ctdnep1_D67E, site-directed mutagenesis was performed by PCR amplification of pDONR221 Ctdnep1 FL vector using primers Ctdnep1_D67E_For and Ctdnep1_D67E_Rev followed by DpnI endonuclease digestion of the parent (methylated) DNA chain. After sequence confirmation, entry vectors were recombined using the Gateway system with pDEST47 (Life Technologies) for C-terminal GFP-Tag fusions proteins and pDEST17 (a gift from Fanny Jaulin Lab) for N-terminal 6xHis-Tag fusion protein. Ctdnep1WT-2xFlag were a gift from Shirin Bahmanyar. Ctdnep1_D67E-2xFlag was generated by site-directed mutagenesis by PCR amplification of Ctdnep1-Flag using the primers hCtdnep1_D67E_For and hCtdnep1_D67_Rev followed by DpnI endonuclease digestion of the parent (methylated) DNA chain.

Human Eps8L2 cDNA and Eps8L2_529-715 were synthetized (Life Technologies) with attB sites to clone it in pDONR201 Gateway donor vector (Life Technologies). The entry vector generated was recombined using the gateway system with pEZYflag (Addgene #18700), pDEST1 or pRK5mycGW (a gift from Fanny Jaulin Lab) to create Flag, GST or Myc N-terminal fusion proteins. Eps8L2 fragments (1-183, 181-299, 297-370, 367-496, 494-546, 544-715) were amplified from Eps8L2 full length using the primers Eps8L2_S1, Eps8L2_R183, Eps8L2_S181, Eps8L2_R299, Eps8L2_S297, Eps8L2_R370, Eps8L2_S367, Eps8L2_R496, Eps8L2_S494, Eps8L2_R546, Eps8L2_S544, Eps8L2_R715 that include attB sites to clone the different fragments in pDONR201 Gateway donor vector (Life Technologies). The entry vectors generated were recombined using the Gateway system with pDEST15 or pRK5mycGW to generate GST or Myc N-terminal fusion proteins. The constructs myc-Eps8L2 and myc-Eps8L2 1_496 used for the dominant negative experiments were synthesized (GenScript) directly into the vector pcDNA3.1+C-MYC. The construct GFP-Eps8L2_1-496 was synthesized (GenScript) directly into the vector pGEX-6P-1. See Table S1 for oligonucleotides information.

For rescue experiments, Ctdnep1-GFP and Ctdnep1_D67E-GFP siRNA resistant sequences adding silent mutations for Ctdnep1 siRNAs #1 and #2 were synthesized (GenScript) and cloned directly to pcDNA3.1(+)-C-eGFP vector.

NLS-GFP (a gift from Jan Lammerding Lab), GFP-Kdel^52^, GFP-miniN2G (a gift from Gregg Gundersen Lab), Lifeact-mcherry (a gift from Olivier Pertz Lab), HaloTag-Sec61β (a gift from Jennifer Lippincott-Schwartz Lab), pGEX-6P-1 (GE Healthcare Life Sciences).

One Shot® TOP10 Chemically Competent *E. coli* cells (Invitrogen) were used to generate all the plasmids.

#### RT-qPCRs for siRNA validation

After 72h transfection with the different siRNAs, total RNA was extracted from fibroblasts cultures using TRIzol® Reagent (ThermoFisher Scientific) according to the manufacturer’s instructions. RNA yield and purity was assessed using Nanodrop 2000 apparatus. cDNA was synthesized using the High capacity RNA-to-cDNA kit (Applied Biosystems) as manufacturer’s instructions indicate. Reverse transcription quantitative PCR (RT-qPCR) was performed using Power SYBR Green PCR MasterMix (Alfagene) according to the manufacturer’s instructions and using primers forward and reverse at 0.25 μM (final concentration) as well as 1:20 cDNA dilution. Amplification of Ctdnep1 mRNA was performed using primers Ctdnep1_Ex3_Ms_For and Ctdnep1_Ex3_Ms_Rev. Amplification of Eps8L2 mRNA was performed using primers Eps8L2 Eps8L2_Ex5_Ms_For and Eps8L2_Ex5_Ms_Rev. As a control, housekeeping gene *gapdh* was amplified using the primers Gapdh_Ms_For and Gapdh_Ms_Rev. RNA extraction, cDNA synthesis and RT-qPCRs were performed three times and relative transcription levels were determined using the Δct method. See Table S1 for oligonucleotides information.

#### siRNA and cDNA infection and microinjection

For NIH 3T3 fibroblasts, the different siRNAs were transfected as previously described^34^ using Lipofectamine RNAiMAX (Invitrogen). Ctdnep1 siRNA #1, Eps8L2 siRNA #1, Eps8L2 siRNA #2 and Eps8L2 siRNA #3 contain Silencer Select modifications (Life Technologies). Ctdnep1 siRNA #2 and Nesprin2G siRNA were from Genecust Europe. Control siRNA (Silencer Select Negative Control Nº 1 siRNA from ThermoFisher Scientific, #4390843). Microinjections for siRNA rescue and immunofluorescence were performed as described in^12^^,^^33^ using a Xenoworks microinjection system (Sutter Instruments). A stable cell line expressing Lifeact-mCherry was created to analyze actin retrograde flow by infecting NIH 3T3 fibroblasts with lentivirus carrying LifeActin-mCherry produced in HEK293T cells (pLALI backbone). Transfected cells were selected by adding puromycin at 2.5 μg/ml during four days.

U2OS and cells SKRB-3 cells were transfected using Lipofectamine 3000 (Invitrogen) with the proper plasmids for 24 hours. After transfection, lysates were obtained for immunoprecipitation.

#### Centrosome reorientation and nuclear movement analysis

Wound assays were performed as described in^33^. In summary, cells were plate on acid-wash coverslips, at the same time that were transfected with the proper siRNA, in a confluence that allows a cell monolayer in the end of the assay. 36-48 hours after transfection (cell confluence around 50%–60%), cells were starved for 48 hours (DMEM with no sodium pyruvate, 10mM HEPES and penicillin/streptomycin at 500 units/ml (Serum Free Medium)). Then, the cell monolayer was scratch-wounded with a pipette tip and cells were stimulated with 10 μM of LPA for 2 hours. Cells were then fix for immunostaining. For TAN lines analysis, stimulation with 10 μM of LPA was performed for 50 minutes. For microinjections, cells were scratch-wounded and microinjected after 48 hours of starvation. Two hours after microinjection, cells were stimulated with 10 μM LPA for 2 hours (nuclear positioning) or 50 minutes (TAN lines and actin dorsal cables thickness). Centrosome and nucleus positions were analyzed using the software *Cell Plot* developed by Gregg Gundersen Lab (^33^ http://www.columbia.edu/∼wc2383/software.html).

#### Cell migration assays

To analyze cell migration during wound closure, wound assays were performed as indicated in the previous section. After 48 hours of incubation with Serum Free Medium, cells were wounded and stimulated with Complete Medium. Imaging of wound closure was performed by acquiring phase-contrast images every 15 minutes for 16 hours.

To analyze single-cell migration (random migration), cells were transfected with siRNAs and grown similar to wound assays. Then, 48 hours after transfection, cells were passed and grown for another 48 hours in order to have a final confluence of 10%–15% approximately, in order to track individual cell migration. Mitomycin C (#sc-3514, Santa Cruz Biotechnology) at 4 μg/mL was added to arrest cell division and phase-contrast images were acquired every 15 minutes for 15 hours.

To quantify instantaneous velocity in wound closure and random migration assays, we manually tracked individual cells (cells at the wound edge for wound closure assays, see Image analysis and figure production section**).**

To analyze the role of Eps8L2_1-496 in cell migration, cells were microinjected after the wound with myc-Eps8L2 or myc-Eps8L2_1-496. Complete Medium were added 2 hours after the microinjection. Cells were fixed and stained after 16 hours. To measure the distance to the wound edge, Fiji software was used to draw a straight line at the leading edge of the most front cells of the wound edge and parallel to the wound scratch. Then, a straight line was drawn from the leading edge of the microinjected cell toward the previous line and perpendicular to it in order to measure the distance.

#### Recombinant protein purification

For His-Ctdnep1_Cter production and purification, pDEST42-Ctdnep1_C-ter plasmid was transformed in Rosetta 2(DE3)pLysS Competent bacteria (Merck Millipore). Bacteria cultures (500 ml) were grown with the proper antibiotics at 20°C until the optical density (OD) was between 0.6 and 0.8. Protein expression was induced by adding 0.4 mM IPTG and the bacteria cultures were grown for 16-20 hours at 16°C. The bacteria culture was centrifuged 15 minutes at 4000 xg and the pellet was resuspended in 20 mL Ctdnep1 Lysis Buffer (50mM Tris-HCl pH 7.5, 200 mM NaCl, lysozyme 0.1 mg/ml, 20 mM imidazole, 1 mM DTT, 0.5 mg/ml Pefabloc® (Roche), 0.5 mM EDTA). The lysate was sonicated on ice for 15 minutes (10 s ON, 10 s OFF) and centrifuged for 30 minutes, 10000 g at 4°C. The supernatant was incubated with 400 μL of Ni-NTA beads (Life Technologies), previously washed with PBS and Lysis Buffer, for 1 hour at 4°C and with rotation. Posteriorly, the beads were centrifuged at 800 g for 4 minutes and washed 2 times with Wash Buffer 1 (50 mM Tris-HCl pH 7.5, 200 mM NaCl, 20 mM Imidazole, and 1 mM DTT). The beads were washed 2 times with Wash Buffer 2 (50 mM Tris-HCl pH 7.5, 1 M NaCl, 30 mM Imidazole). The beads were kept in PBS, 1 mM DTT and 40% glycerol at −80°C. To elute His-Ctdnep1_C-ter protein, the beads were incubated for 2 hours at 4°C in Elution Buffer (50 mM Tris-HCL pH 7.5, 200 mM NaCl, 400 mM Imidazole and 1 mM DTT). The eluted fraction was concentrated using a 3 KDa MWCO Amicon® Ultra-15 Centrifugal Filter Unit using Stockage Buffer (50 mM Tris-HCl pH 7.5 and 200 mM NaCl).

For GST, GST-Eps8L2 and GST-Eps8L2 fragments, pDEST15 plasmids were transformed in Rosetta 2(DE3)pLysS Competent bacteria (Merck Millipore). Bacteria cultures (1 Liter) were grown with the proper antibiotic at 37°C until an OD of 0.6 was obtained. Protein expression was induced adding 0.1 mM IPTG and incubate for 4 hours at 34°C. The bacteria pellet was recovered by centrifuging 15 minutes at 4000 xg. The pellet was lysed with 20 mL (per 250ml of bacteria culture) of Eps8L2 Lysis Buffer (10 mM Tris-HCL pH8, 150 mM NaCl, 1 mM EDTA, lysozyme 0.1 mg/ml, Pefabloc® (Roche) 0.5 mg/ml) for 30 minutes at 4°C to dissolve the pellet. DTT (1 mM) and Sarkosyl (N-Lauroylsarcosine sodium salt solution 1.4%, dissolved in Eps8L2 Lysis Buffer without lysozyme nor Pefabloc® (Roche)) were added posteriorly. The lysate was sonicated on ice for 15 minutes (10 s ON, 10 s OFF) and centrifuged for 45 minutes at 39000 xg and 4°C. The supernatant was collected and incubated with 20 mL of Eps8L2 Lysis Buffer without lysozyme nor Pefabloc® (Roche), and 4% Triton X-100 for 30 minutes at 4°C. The lysate was then incubated with Glutathione Sepharose 4B beads (GE Healthcare Life Sciences) previously washed 3 times with cold PBS. The incubation was for 2 hours at 4°C with rotation. Then, the beads were centrifuged for 4 minutes at 800 g and washed three times with cold PBS. The beads were kept in PBS, 1 mM DTT and 40% glycerol at −80°C. It is important to mention that the fragment GST-Eps8L2_297-370 was not possible to purify due to its insolubility.

To check the purification process, an aliquot of each step was taken to run a SDS-Page for BlueSafe (NZYTech #MB15201) staining or Western Blot.

#### Immunoprecipitation

For *in vitro* Pull Down, 20 μg of GST and GST-Eps8L2 beads (completed with Glutathione Sepharose 4B beads until 30 μL of total beads if needed) were washed with Wash/Binding Buffer (125 mM Tris-HCl, 150 mM NaCl). The beads were incubated with equal amount of eluted His-Ctdnep1_C-ter (final volume of 300 μL in Wash/Binding Buffer) for 3 hours at 4°C with rotation. The beads were posteriorly washed (by centrifuging at 800 g for 5 minutes) three times with Wash/Binding Buffer. All supernatant was removed using a 1 mL syringe and the beads were resuspended in 30 μL 2X SDS Sample Buffer (Merck Millipore). The samples were incubated at 98°C for 5 minutes before electrophoresis.

For the Eps8L2 fragments Pull Down, 20 μg of GST, GST-Eps8L2 and GST-Eps8L2 fragments proteins bound to glutathione beads (complete with Glutathione Sepharose 4B beads until 30 μL of total beads if needed) were washed with Lysis Buffer (25 mM Tris-HCl, 100 mM NaCl, 1% Triton X-100, 10% Glycerol). To make GFP-KDEL, Ctdnep1-GFP and Ctdnep1_D67E-GFP lysates, U2OS cells were transfected with the plasmids and lysates were made using U2OS Lysis Buffer. The beads were incubated with the lysates (600 μg total protein, complete till 300 μL with Lysis buffer) for 3 hours at 4°C with rotation. Posteriorly, the beads were washed (by centrifuging at 800 g for 5 minutes) three times with IP1500 Buffer (50 mM Tris-HCl pH 7.5, 50 mM NaCl, 0.1% Triton X-100). All supernatant was removed using a 1 mL syringe and the beads were resuspended in 30 μL 2X SDS Sample Buffer (Merck Millipore). The samples were incubated at 98°C for 5 minutes before electrophoresis.

Co-Immunoprecipitation of Ctdnep1 and endogenous Eps8L2 was performed by using SKBR-3 cells (high amount of endogenous Eps8L2 expression). SKBR-3 cells were incubated with SKBR Lysis Buffer (20 mM HEPES pH 7, 10 mM KCl, 0.1% NP40, 6 mM MgCl2, 20% glycerol) on ice for 10 minutes to posteriorly collect the lysates. Lysates were sonicated for 15 s and 10 mA and they were incubated with 5U of DNaseI 60 min at 4°C. For pre-clearing, lysates were incubated with 10 μL of Protein A/G magnetic beads (Life Technologies) for 30 minutes at 4°C and rotation. The beads were collected using a magnetic rack. SKBR-3 cell lysates were mixed equally (1 mg total protein) to lysates from U2OS cells overexpressing NLS-GFP, Ctdnep1-GFP or Ctdnep1_D67E-GFP and incubated with Protein A/G magnetic beads and 2.8 mg of Eps8L2 antibody in IP Buffer (500ul total volume, 20 mM HEPES pH 7, 10 mM KCl, 1.5 mM MgCl2, 0.2% Tween20, 10% glycerol, 1 mM DTT). Incubation was performed overnight at 4°C. The beads were collected using a magnetic rack and washed with Wash Buffer 1 (20 mM HEPES pH 7, 50 mM KCl, 1.5 mM MgCl2, 0.2% Tween20, 10% glycerol, 1 mM DTT), Wash Buffer 2 (20 mM HEPES pH 7, 100 mM KCl, 1.5 mM MgCl2, 0.2% Tween20, 10% glycerol, 1 mM DTT) and Wash Buffer III (20 mM HEPES pH 7, 150 mM KCl, 1.5 mM MgCl2, 0.2% Tween20, 10% glycerol, 1 mM DTT). Finally, the beads were resuspended in 30 μL 2X SDS Sample Buffer (Merck Millipore) and incubated at 55°C for 5 minutes before electrophoresis.

To immunoprecipitate myc-Eps8L2 for the phosphorylation assay, 500 μL (30 μg/μl total protein) of the different lysates were pre-cleared with 20 μL of control agarose beads previously washed two times with PBS and once with U2OS Lysis Buffer. The lysates were collected by centrifuging at 16000 g for 10 minutes and incubated with 50 μL of Myc-Trap®_A beads (Chromotek #yta-20, previously washed as before) for 2 hours at 4°C with rotation. The beads were washed three times with IP1500 buffer. The supernatant was removed with a 1 mL syringe and the beads were resuspended in 30 μL 2X SDS Sample Buffer (Merck Millipore). The samples were incubated at 98°C for 5 min and the samples were posteriorly collected by centrifuging for 5 minutes at 16000 xg.

Yeast Two-Hybrid assay was performed by Hybrigenics using Ctdnep1_Cter as bait fragment, Human Placenta_RP5 as prey library and pB27 (N-LexA-bait-C fusion) as vector.

#### Phosphorylation analysis

To analyze Eps8L2 phosphorylation state, the samples were run in SuperSep™ Phos-tag™ 7.5% (Wako Chemicals) following manufacturer’s instructions. As a control, the same samples were run in parallel in a Mini-PROTEAN® TGX 4%–15% Precast protein gels (BioRad). For mass spectrometry assay, U2OS cells were transfected with myc-Eps8L2 and co-transfected with Ctdnep1-GFP or Ctdnep1_D67E-GFP. Lysates were obtained as indicated in immunoprecipitations section. Immunoprecipitations were performed in three independent experiments and all the samples were send to Proteomics Core Facility at EMBL (Heidelberg). TMT labeling for the individual samples was performed according to the manufacturer’s instructions. Samples preparation before mass spectrometry to identify and quantify Eps8L2 phosphopeptides was performed according to previous work^53^.

#### Western blots

Protein samples were run in Mini-PROTEAN® TGX 4%–15% Precast protein gels (BioRad) and transferred to nitrocellulose membranes. Western blots were probed using the following primary and secondary antibodies (incubated in PBS, 0.1% Tween20 and 5% milk): rabbit anti-Eps8L2 (Sigma-Aldrich #HPA041143, 1:500), mouse anti-Flag (Sigma-Aldrich #F1804, 1:1000), mouse anti-6X His (Abcam #ab18184, 1:1000), chicken anti-GFP (Aves Labs #GFP-1020, 1:2000), anti-mouse HRP (Thermo Scientific #32430, 1:5000), anti-rabbit HRP (Thermo Scientific #31460, 1:5000), anti-chicken HRP (Jackson ImmunoResearch Laboratories # 703-035-155, 1:5000).

#### Immunofluorescence

Cells were fixed in 4% paraformaldehyde in PBS for 10 minutes at room temperature. Permeabilization with 0.3% Triton X-100 for 5 minutes or 40 μg/ml Digitonin for 3 min at room temperature were posteriorly performed. Primary and secondary antibodies were diluted in PBS containing 10% goat serum. Cells were incubated with primary antibodies for 1 hour at room temperature. After washing three times (20 minutes each) with PBS, cells were incubated with secondary antibodies for 1 hour at room temperature. Cells were washed with PBS (3 times, 20 minutes each time). Fluoromount-G (Invitrogen) was used to prepare the coverslips for cell imaging. The following primary antibodies were used: rabbit anti-β-Catenin 1:200 (Invitrogen #712700), mouse anti-Pericentrin 1:200 (BD-Biosciences # 611814), mouse anti-Flag 1:200 (Sigma-Aldrich #F1804), chicken anti-GFP 1:1000 (Aves Labs #GFP-1020), rabbit anti-LaminB1 1:200 (Abcam #ab16048), mouse anti-Myc 1:200 (Alfagene/Life Technologies #13-2500), mouse anti-Vinculin 1:200 (Sigma-Aldrich V9131). The secondary antibodies (1:600 dilution) used were Alexa Fluor 488, Alexa Fluor 555 and Alexa Fluor 647 (Life Technologies). Phalloidin conjugated with Alexa Fluor 488 and Alexa Fluor 647 (Life Technologies) were used to stain actin (1:200 dilution). DAPI (Sigma-Aldrich) was used to stain the nucleus (1:10000 dilution).

For ER staining, cells were incubated with Janelia Fluor® 646 HaloTag® (Promega, GA1120) for 30 minutes following the manufacturer’s instructions. Cells where then fixed with methanol at −20°C for 10 minutes. Then, cells were washed with PBS (3 times, 1 minute each time) and stained as shown previously but without Triton or Digitonin permeabilization.

#### Cell imaging

For centrosome reorientation images were acquired in a Zeiss Cell Observer widefield inverted microscope equipped with sCMOS camera Hamamatsu ORCA-flash4.0 V2 for 10ms/frame streaming acquisition, EC Plan-Neofluar 40x/0.75 M27 Oil Objective, LED light source Colibri2 from Zeiss, FS38HE excitation 450-490 nm and emission 500-550 nm and FS43HE excitation 538-562 nm and emission 570-640 nm controlled by with the ZEN Blue Edition software. For TAN lines and actin thickness quantification the same microscope was used although with a 63x/1.4 Plan-Apochromat DIC M27 Oil objective. For wound closure assays with the Eps8L2 dominant negative construct, the same microscope was used with a 20x/0.8 Plan-Apochromat Ph2 dry objective. Actin retrograde flow analysis was performed using a Zeiss Cell Observer spinning disk confocal inverted microscope equipped with a 37°C chamber, 5% CO_2_ live-cell imaging chamber, Evolve 512 EMCCD camera, confocal scanner Yokogawa CSU-x1, 63x Plan-Apochromat Oil Objective, LED light source Colibri2 from Zeiss, solid state laser 405 nm and 561 nm controlled by ZEN Blue Edition. Confocal images for protein localization at TAN lines were acquired using a Zeiss LSM 710 microscope equipped with a 63 /1.4 Plan-Apochromat DIC M27 Oil objective, diode laser 405-30 (405 nm), Argon laser (458, 488 and 514 nm), DPSS 561-10 laser (561 nm) and HeNe633 laser (633 nm).

For wound closure assays, images were acquired using a Nikon Ti microscope equipped with a heated chamber at 37°C with 5% CO_2_ (Okolab) and a 10x/0.30 CFI Plan Fluor DLL Ph1 objective (Nikon). Phase-contrast images were collected with a Retiga R6 mono CCD camera (Teledyne QImaging) using the Perfect Focus software controlled by Metamorph software (Molecular Devices).

For random migration assays, images were acquired using a Zeiss Axio Observer microscope equipped with a heated chamber at 37°C with 5% CO_2_ and a 10x/0.30 EC Plan-Neofluar M27 Ph1 objective (Zeiss). Phase-contrast images were collected with an Axiocam 506 mono CCD camera controlled by ZEN 2 Blue Edition software.

#### Dorsal actin cables measurement

The number of dorsal actin cables over the nucleus was obtained counting all the dorsal actin cables observed on top of the nucleus. We used DAPI staining to delimit the nuclear region and Phalloidin to observed actin cables.

Actin thickness of dorsal actin cables and actin cables at the leading edge was performed as indicated in Janota et al., 2020^54^. Actin cables were identified by Phalloidin staining. Three dorsal actin cables (or two if that was the maximum number of dorsal actin cables) and three actin cables at the leading edge were quantified per cell. Briefly, to quantify the thickness of one actin cable, a straight line was drawn perpendicular to the actin cable to posteriorly obtain the plot profile of the fluorescence intensity along the line using Fiji software. The two bottom points of the curve observed (when the slope of the curve starts to decidedly increase) represent the actin cable beginning and end. The distance between these two points represents the actin cable length.

#### TAN lines protein localization measurement

Quantification of protein localization at TAN lines was performed as previously shown in Saunders et al., 2017^55^. Briefly, TAN lines images were rotated in order to obtain the TAN line parallel to the x axis. An 11-pixel-tall box was drawn to cover the length of the TAN line with the central pixel centered on the TAN line. The mean intensity for each row of pixels was calculated for every channel separately to posteriorly express each mean intensity as a percentage of the sum intensity of the whole measured region. The regions were then mirrored by taking the means of the 1^st^ and 11^th^, 2^nd^ and 10^th^, etc. pixel. After quantifying all TAN lines, fractional fluorescence of each pixel row was then calculated and the mean and SEM were plotted as a function of the distance from the center of the TAN lines.

#### Image analysis and figure production

Fiji software was used as an imaging processing software, to quantify actin filaments thickness and wound closure velocity. For instantaneous velocity in random cell migration and wound closure, individual cells were then tracked using Fiji with the Manual Tracking plug-in. For wound closure assays, only cells at the wound edge were tracked. The average instantaneous velocity and directionality for each cell was obtained through the Chemotaxis and Migration Tool (https://ibidi.com/chemotaxis-analysis/171-chemotaxis-and-migration-tool.html).

#### Directionality was expressed as

$$Di=\frac{di,\;euclidean}{di,\;accumulated}$$

Where di,euclidean is the Euclidean distance and di,accumulated is the accumulated distance.

Wound closure velocity was quantified by measuring the wound area at 0 and 16 hours and calculating wound closure velocity according to the following formula:

$$v\;\left(\mu m/min\right)=\frac{\Delta A}{2\times y\times \Delta t}$$

Where, ΔA is the wound area closed by cells after 16 hours, *y* is the wound height and Δt is the duration of the assay (960 minutes).

We used the data obtained from the manual tracking to analyze the angles of migration during wound closure. Absolute angles were calculated with the R package, amt. We started by removing the time points where x and y coordinates where identical to the previous step. Then each track was converted to the basic building block of the *amt* package^56^ through the function *make_track()*. Lastly, we applied the functions *direction_abs()* to get the absolute angles for each segment of the track. Absolute angles were calculated clockwise and between ± π. An absolute angle of 90° means the cell migrates perpendicular to the wound edge. As a readout of migration directionality and persistence, we quantified total distribution of the absolute angles as well as percentage of absolute angles per cell centered at 90° (between 67.5° and 112°).

To quantify Eps8L2-Ctdnep1 colocalization, we selected a squared ROI with fixed dimensions (10x10μm) for the cell front (near the leading edge) whereas for the perinuclear region, we chose a circular ROI that encompasses 3 μm from the nuclear envelope (using DAPI staining to determine the shape of the nucleus). Colocalization was performed by measuring the Pearson correlation coefficient and calculating the Manders overlap coefficient implemented in the FIJI plug-in, *JACOP*. For the Manders overlap coefficient, we applied a manual threshold on both channels and we considered Image A to be our myc-Eps8L2 channel (Red) and Image B to be our Ctdnep1-GFP or Ctdnep1_D67E-GFP channel (Green). Our quantification was based on the M1 coefficient, corresponding to fraction of summed red pixels for which green intensity is greater than zero, to the total intensity in the red channel.

For Vinculin quantification, images were enhanced by applying a difference of Gaussian (DoG) filter. A manual threshold was used to binarize objects in Vinculin images. These objects were automatically identified using ImageJ Analyze Particles function, where we imposed morphological restrictions to filter out small round objects originated by residual noise. We measured the number and the area of segmented objects per cell.

Adobe Photoshop and Adobe Illustrator were used to generate figures.

### Quantification and statistical analysis

The number of experiments and cells quantified are indicated in individual Figure Legends and plots. Graphpad Prism 8 software was used to analyze and represent the data. Results are expressed as mean ± SEM for bars plots. Statistical significance was assessed using unpaired t tests: ^∗^p < 0.05, ^∗∗^p < 0.01, ^∗∗∗^p < 0.001, ^∗∗∗∗^p < 0.0001.

## Supplemental information

Document S1. Figures S1–S4 and Table S1

Document S2. Article plus supplemental information
