# Supplementary material for: Ctdnep1 and Eps8L2 regulate dorsal actin cables for nuclear positioning during cell migration
Source: Curr Biol. 2021 Apr 12;31(7):1521–1530.e8. doi: 10.1016/j.cub.2021.01.007 (PMC8043254; doi:10.1016/j.cub.2021.01.007)
Supplement: Document S1. Figures S1–S4 and Table S1 [file mmc1.pdf]

Current Biology, Volume *31*

Supplemental Information

Ctdnep1 and Eps8L2 regulate dorsal actin cables

for nuclear positioning during cell migration

Francisco J. Calero-Cuenca, Daniel S. Osorio, Sofia Carvalho-Marques, Sreerama Chaitanya Sridhara, Luis M. Oliveira, Yue Jiao, Jheimmy Diaz, Cátia S. Janota, Bruno Cadot, and Edgar R. Gomes

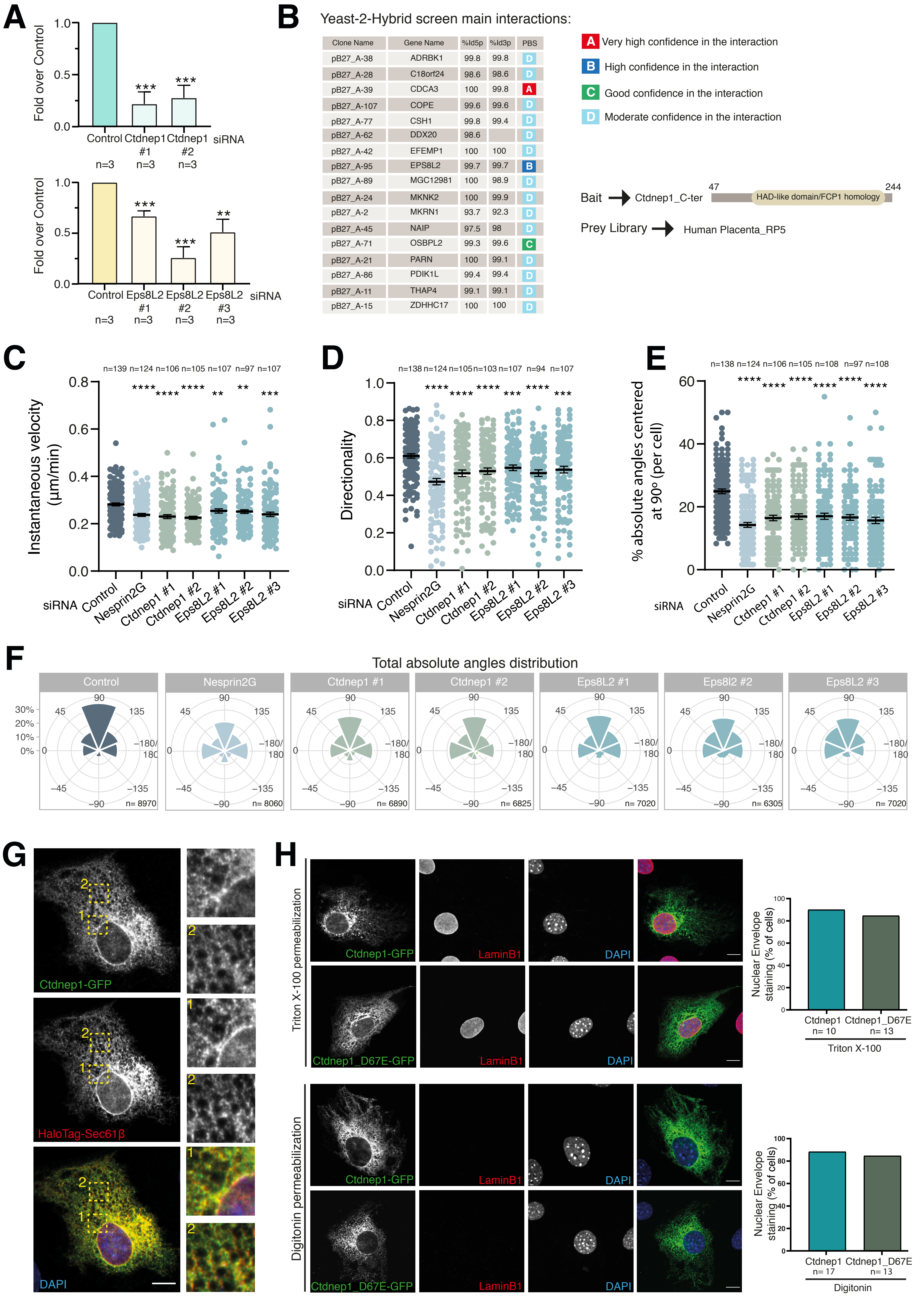

Clone Name	Gene Name	%Id5p	%Id3p	PBS
pB27_A-38	ADRBK1	99.8	99.8	D
pB27_A-28	C18orf24	98.6	98.6	D
pB27_A-39	CDCA3	100	99.8	A
pB27_A-107	COPE	99.6	99.6	D
pB27_A-77	CSH1	99.8	99.4	D
pB27_A-62	DDX20	98.6		D
pB27_A-42	EFEMP1	100	100	D
pB27_A-95	EPS8L2	99.7	99.7	B
pB27_A-89	MGC12981	100	98.9	D
pB27_A-24	MKNK2	100	99.9	D
pB27_A-2	MKRN1	93.7	92.3	D
pB27_A-45	NAIP	97.5	98	D
pB27_A-71	OSBPL2	99.3	99.6	C
pB27_A-21	PARN	100	99.1	D
pB27_A-86	PDIK1L	99.4	99.4	D
pB27_A-11	THAP4	99.1	99.1	D
pB27_A-15	ZDHHC17	100	100	D

Figure S1. Depletions of Ctdnep1 and Eps8L2 affect cell migration and directionality. Related to Figure 1. (**A**) Ctdnep1 mRNA quantification (top) in 3T3 fibroblasts transfected with Control, Ctdnep1 #1 and Ctdnep1 #2 siRNAs measured by RT-qPCR. Eps8L2 mRNA quantification (bottom) in 3T3 fibroblasts transfected with Control, Eps8L2 #1, Eps8L2 #2 and Eps8L2 #3 siRNAs measured by RT-qPCR. (**B**) Yeast Two-Hybrid screening performed with Ctdnep1_C-ter as the bait and human placenta library as prey. The table shows the main interactions observed in the screening. Each interaction presents a Predicted Biological Score (PBS) that is computed to assess the interaction reliability. This score represents the probability of an interaction to be non-specific: it is an e-value, primarily based on the comparison between the number of independent prey fragments found for an interaction and the chance of finding them at random (background noise). %Id5p and %Id3p indicates the % identity of the prey fragment sequences with the gene reference sequence. Average instantaneous velocity (**C**) and directionality (**D**) during wound closure in cells at the wound edge treated with Control, Nesprin2G, Ctdnep1 and Eps8L2 siRNAs. (**E**) Percentage of angles per cell with a value between 67.5^{o} and 112° (angles centered at 90°) relative to the wound edge (absolute angle) during wound closure assays in cells treated with Control, Nesprin2G, Ctdnep1 and Eps8L2 siRNAs. (**F**) Total absolute angles (relative to the wound edge) distribution in wound closure assays in cells treated with Control, Nesprin2G, Ctdnep1 and Eps8L2 siRNAs. (**G**) Representative images of wound-edge wildtype fibroblasts stimulated with LPA and microinjected with Ctdnep1-GFP and HaloTag-Sec61β stained for GFP (green), HaloTag (red, endoplasmic reticulum) and Dapi (blue, nucleus). The dashed yellow squares indicate the region of the insets shown in the right panels. (**H**) Representative image of wound-edge fibroblasts stimulated with LPA and microinjected with Ctdnep1-GFP or Ctdnep1_D67E-GFP fixed and permeabilized with Triton X-100 or digitonin and stained for GFP (green), LaminB (red) and DAPI (blue, nucleus). Plots indicating the quantification of nuclear envelope staining are shown on the right. Triton treatment permeabilizes the plasma membrane and nuclear envelope whereas digitonin only permeabilizes the plasma membrane and not the nuclear envelope. Scale bar: 10 µm. Data are represented as mean +/- SEM in A, C, D and E. The n value means number of experiments (A), number of analysed cells (C, D, E and H) or number of total angles analysed (F). Statistics was performed by unpaired t test: *$p<0.05$, **$p<0.01$, ***$p<0.001$, ****$p<0.0001$.

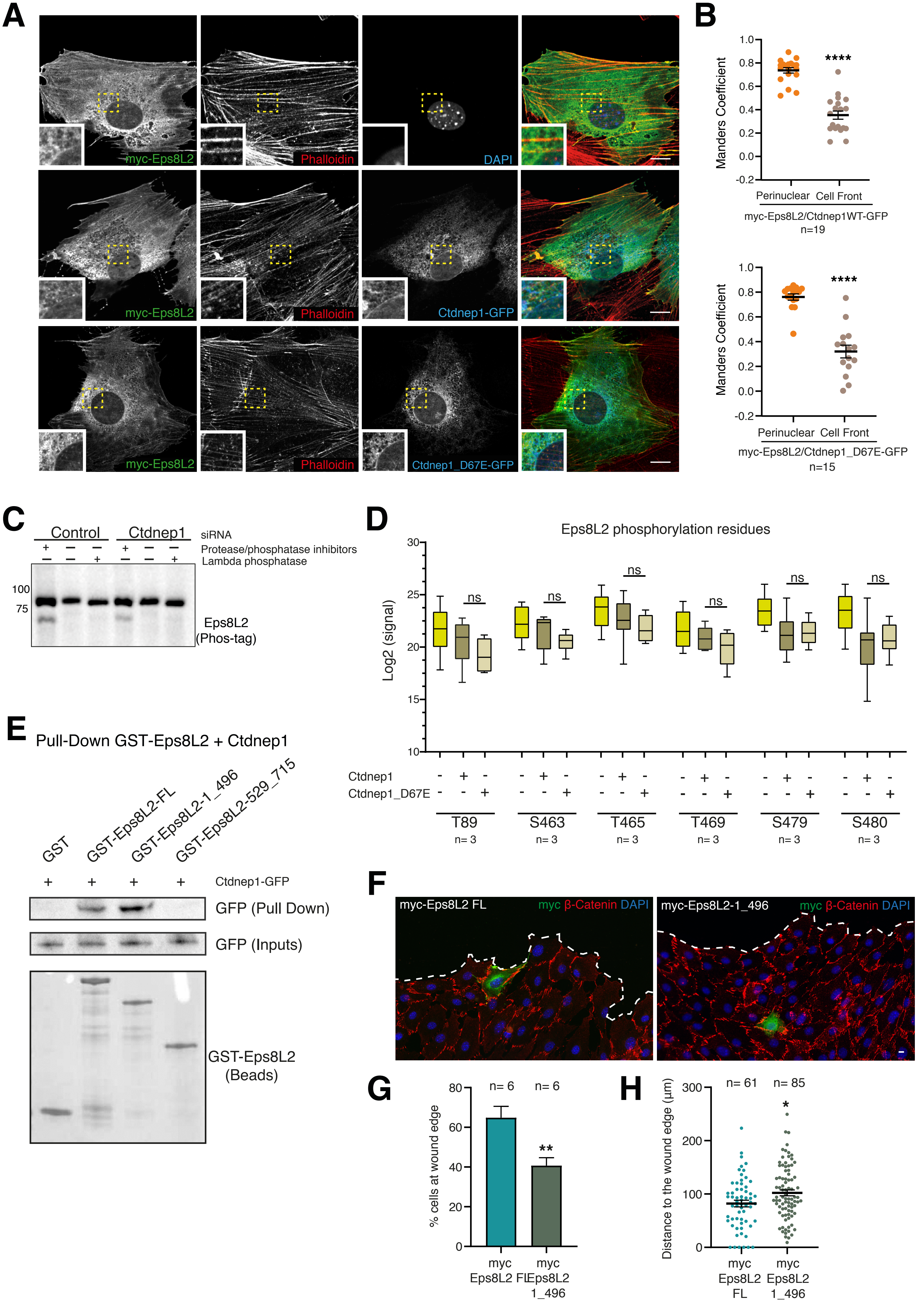

A
myc-Eps8L2
Phalloidin
DAPI
Ctdnep1-GFP
Ctdnep1_D67E-GFP
B
Manders Coefficient
****
Perinuclear
Cell Front
myc-Eps8L2/Ctdnep1WT-GFP
n=19
myc-Eps8L2/Ctdnep1_D67E-GFP
n=15
C
Control
Ctdnep1
siRNA
Protease/phosphatase inhibitors
Lambda phosphatase
100
75
Eps8L2
(Phos-tag)
D
Eps8L2 phosphorylation residues
Log2 (signal)
ns
Ctdnep1
Ctdnep1_D67E
T89
S463
T465
T469
S479
S480
n= 3
E
Pull-Down GST-Eps8L2 + Ctdnep1
GST
GST-Eps8L2-FL
GST-Eps8L2-1_496
GST-Eps8L2-529_715
Ctdnep1-GFP
GFP (Pull Down)
GFP (Inputs)
GST-Eps8L2
(Beads)
F
myc-Eps8L2 FL
myc β-Catenin DAPI
myc-Eps8L2-1_496
G
% cells at wound edge
n= 6
**
myc
Eps8L2 FL
Eps8L2
1_496
H
Distance to the wound edge (μm)
n= 61
n= 85
*
myc
Eps8L2
FL
1_496

Figure S2. Ctdnep1-Eps8L2 interaction regulates cell migration. Related to Figure 2. (**A**) Representative images of wound-edge wildtype fibroblasts stimulated with LPA and microinjected with myc-Eps8L2, Ctdnep1-GFP and Ctdnep1_D67E-GFP stained for myc (green, Eps8L2), Phalloidin (red, Actin) DAPI (blue, nucleus) or GFP (blue, Ctdnep1 and Ctdnep1_D67E). Dashed yellows squares indicate the regions of the insets shown in the bottom left corner. (**B**) Quantification of Eps8L2 and Ctdnep1 colocalization by analysing the Manders Coefficient in the conditions shown in A and in the perinuclear region (3 µm from the nucleus border) or in the front cell (close to the leading edge). The n value means number of analysed cells. (**C**) Phos-tag gel for endogenous Eps8L2 in SKBR-3 cells (that express high levels of Eps8L2) treated with Control and Ctdnep1 siRNAs. Incubation with Lambda phosphatase was used as a control to remove all phosphorylation in the sample. (**D**) Plot representing the Log_2 of the signal obtained in the mass spectrometry analysis for each phosphorylated residues detected in Eps8L2 with or without co-transfection with Ctdnep1 and Ctdnep1_D67E. The plot shows the results from three independent experiments (n). (**E**) Pull Down assay of recombinant GST, GST-Eps8L2-FL, GST-Eps8L2-1_496 and GST-Eps8L2-529_715 proteins bound to glutathione agarose beads with Ctdnep1-GFP overexpressed in U2OS cells. (**F**) Representative images of wound closure assays in cells microinjected with myc-Eps8L2 or myc-Eps8L2_1-496 and stained for myc (green), β-catenin (red, cell contacts) and DAPI (blue, nucleus). The dashed white lines mark the wound edge. (**G**) Quantification of percentage of cells at the wound edge in the wound closure experiments shown in E. Data are represented as mean +/- SEM. The n value means number of experiment (>10 cells per experiment). (**H**) Quantification of average distance to the wound edge in the wound closure in the conditions shown in E. The n value means number of analysed cells. Scale bars: 10 µm. Statistics was performed by unpaired t test: *$p<0.05$, **$p<0.01$, ***$p<0.001$, ****$p<0.0001$.

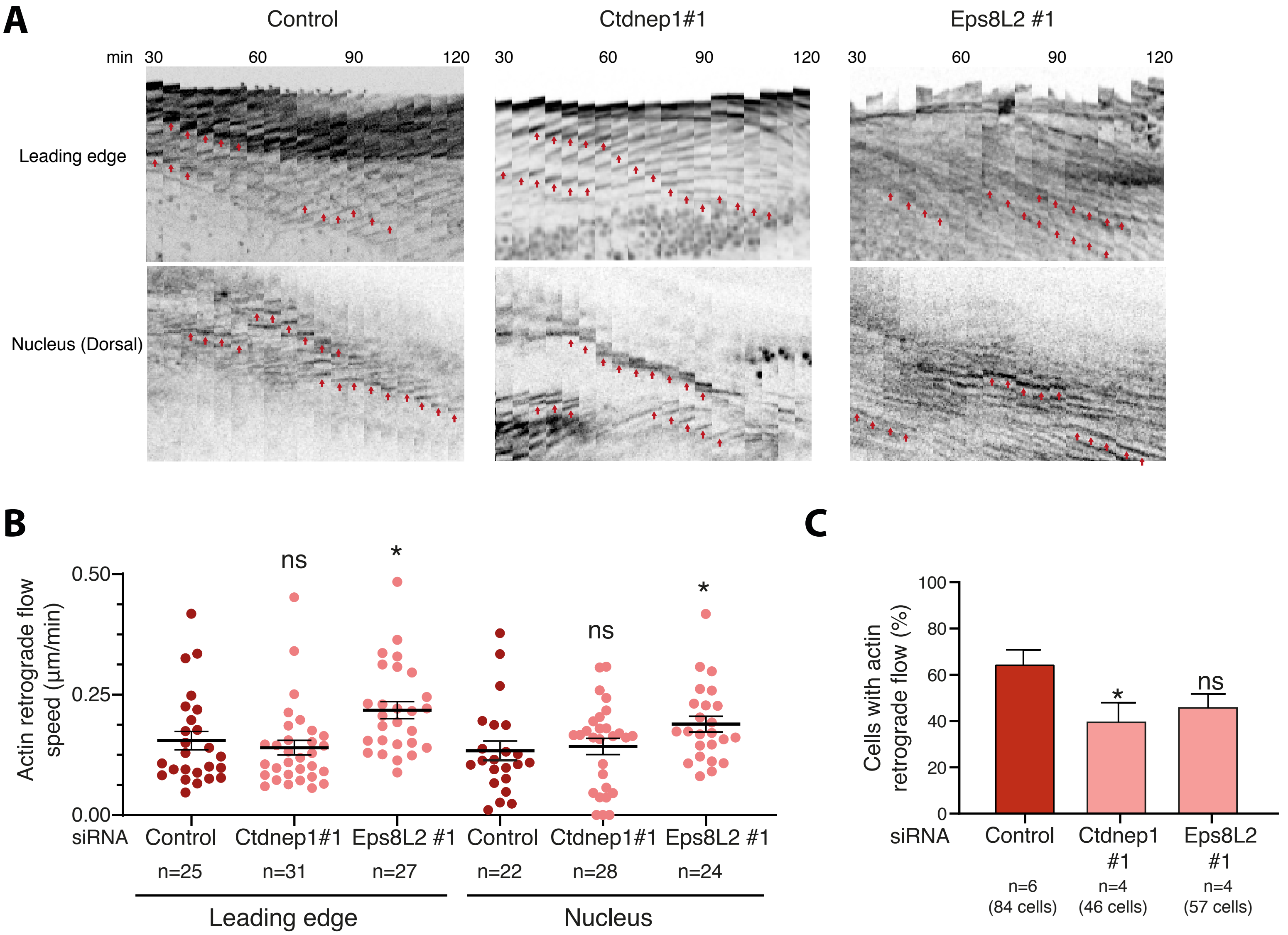

A
Control
Ctdnep1#1
Eps8L2 #1
min 30 60 90 120
30 60 90 120
30 60 90 120
Leading edge
Nucleus (Dorsal)
B
Actin retrograde flow speed (µm/min)
0.50
0.25
0.00
ns
*
ns
*
siRNA
Control
Ctdnep1#1
Eps8L2 #1
Control
Ctdnep1#1
Eps8L2 #1
n=25
n=31
n=27
n=22
n=28
n=24
Leading edge
Nucleus
C
Cells with actin retrograde flow (%)
100
80
60
40
20
0
*
ns
siRNA
Control
Ctdnep1 #1
Eps8L2 #1
n=6 (84 cells)
n=4 (46 cells)
n=4 (57 cells)

Figure S3. Actin retrograde flow is not strongly impaired by Ctdnep1 or Eps8L2 depletions. Related to Figure 3. (**A**) Kymographs of the actin retrograde flow at the leading edge (top panels) or on top of the nucleus (bottom panels) in wound-edge fibroblasts after LPA stimulation and treated with Control, Ctdnep1 or Eps8L2 siRNAs. A stable cell line of 3T3 fibroblasts expressing Lifeact-mcherry were used to measure actin retrograde flow. The red arrows indicate the movement of the actin cables. (**B**) Quantification of actin retrograde flow speed for actin filaments located near the leading edge (Leading edge) or on the dorsal side of the nucleus (Nucleus) in the conditions showed in A. **(C)** Quantification of the percentage of wound-edge fibroblasts with actin retrograde flow in the conditions shown in A. Data are represented as mean +/- SEM. The n value means number of cells (B) or experiments (C). Statistics was performed by unpaired t test: *$p<0.05$, **$p<0.01$, ***$p<0.001$, ****$p<0.0001$.

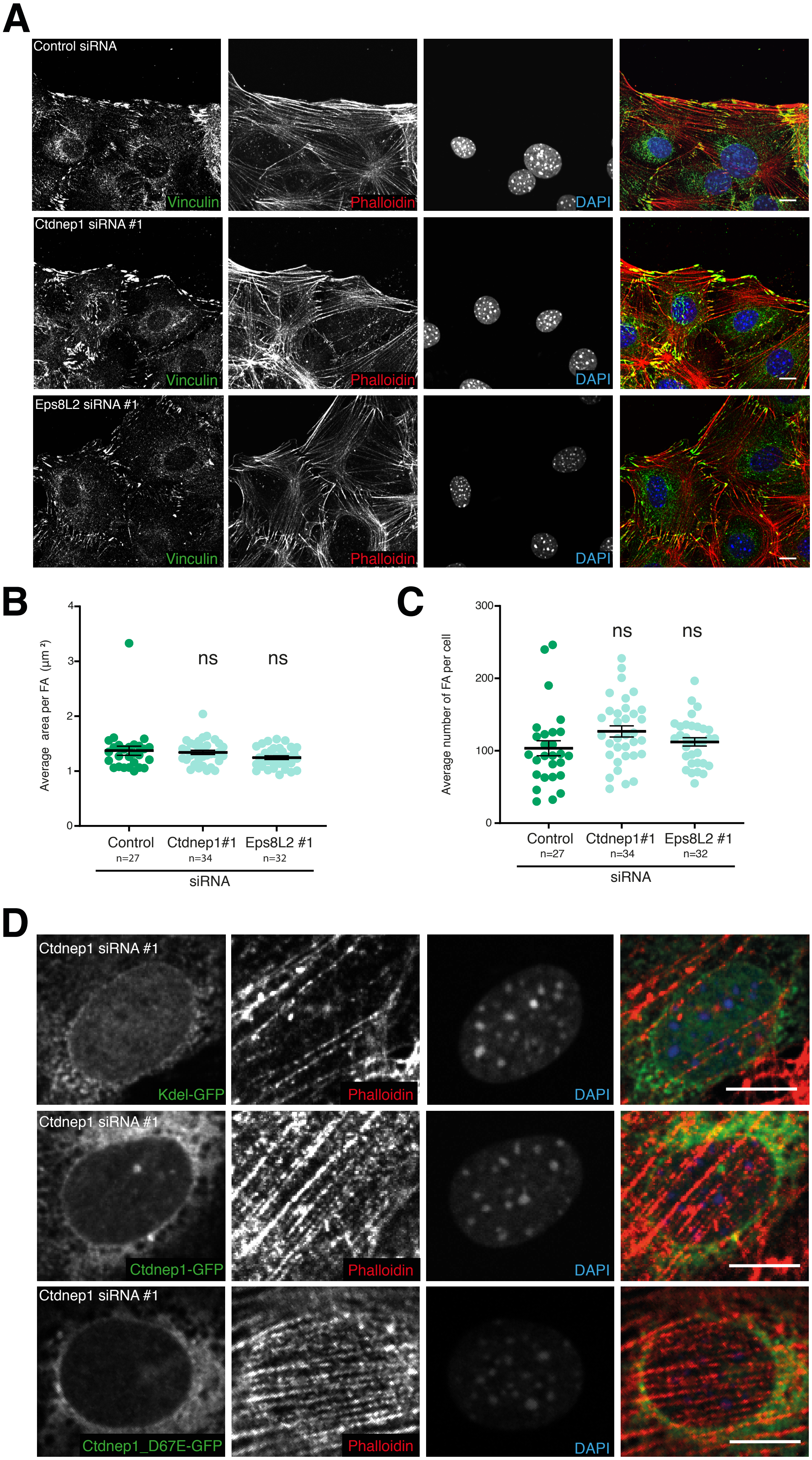

A
Control siRNA
Vinculin
Phalloidin
DAPI
Ctdnep1 siRNA #1
Vinculin
Phalloidin
DAPI
Eps8L2 siRNA #1
Vinculin
Phalloidin
DAPI
B
Average area per FA (μm²)
0
1
2
3
4
ns
ns
Control
n=27
Ctdnep1#1
n=34
Eps8L2 #1
n=32
siRNA
C
Average number of FA per cell
0
100
200
300
ns
ns
Control
n=27
Ctdnep1#1
n=34
Eps8L2 #1
n=32
siRNA
D
Ctdnep1 siRNA #1
Kdel-GFP
Phalloidin
DAPI
Ctdnep1 siRNA #1
Ctdnep1-GFP
Phalloidin
DAPI
Ctdnep1 siRNA #1
Ctdnep1_D67E-GFP
Phalloidin
DAPI

Figure S4. Ctdnep1 or Eps8L2 depletions do not affect overall actin organization. Related to Figure 4. (**A**) Representative images of wound-edge fibroblasts stimulated with LPA and treated with Control, Ctdnep1 and Eps8L2 siRNAs. Cells were stained for Vinculin (focal adhesions, green), Phalloidin (actin, red) and DAPI (nucleus, blue). (**B**) Quantification of average Vinculin area per focal adhesion analysed in the conditions shown in A. (**C**) Quantification of average number of focal adhesions analyzed in the conditions shown in A. Data are represented as mean +/- SEM. The n value means the number of cells. (**D**) Representative images of the nucleus of wound-edge fibroblasts stimulated with LPA, treated with Ctdnep1 siRNA and microinjected with KDEL-GFP, Ctdnep1-GFP and Ctdnep1D67E-GFP. Cells were stained for GFP (green), phalloidin (actin, red) and DAPI (nucleus, blue). Scale bars: 10 µm. Statistics was performed by unpaired t test: *p<0.05, **p<0.01, ***p<0.001, ****p<0.0001.

REAGENT or RESOURCE	SUPPLIER	IDENTIFIER
**Oligos used for plasmids cloning**		
5’-GGGGACAAGTTTGTACAAAAAAGCAGGCTTAAGGAGGTACCATGATGCGGACGCAGTGT-3’	Sigma-Aldrich	Ctdnep1_FL_N1_For
5’-GGGGACCACTTTGTACAAGAAAGCTGGGTCCCAGAGCCTATGTTGGTGAAG-3’	Sigma-Aldrich	Ctdnep1_FL_N1_Rev
5’-GGGGACAAGTTTGTACAAAAAAGCAGGCTTACCCTTATCTCCTTTGTCC-3’	Sigma-Aldrich	Ctdnep1_Cter_C1_For
5´-GGGGACCACTTTGTACAAGAAAGTGGGTCTCACCAGAGCCTATGTTGGTG-3’	Sigma-Aldrich	Ctdnpe1_FL_C1_Rev
5’-GATCCTGGTGCTGGAACTGGACGAAACCCTG-3’	Sigma-Aldrich	Ctdnep1_D67E_For
5’-CAGGGTTTCGTCCAGTTCCAGCACCAGGATC-3’	Sigma-Aldrich	Ctdnep1_D67E_Rev
5’-GATCCTGGTGCTGGAGCTGGATGAGACAC-3’	Sigma-Aldrich	hCtdnep1_D67E_For
5’-GTGTCTCATCCAGCTCCAGCACCAGGATC-3’	Sigma-Aldrich	hCtdnep1_D67E_Rev
5’-CCCCGGACTGGCTCATGGAGCCTGCTTTTTTGTACAAACTTGTCCCC-3’	Sigma-Aldrich	Eps8L2_S1
5’-AAGATGCGGCCGCAGTAGGACCCAGCTTTCTTGTACAAAGTGGTCCCC-3’	Sigma-Aldrich	Eps8L2_R183
5’-CAGGGTCTGCGGCCGGGAGCCTGCTTTTTTGTACAAACTTGTCCCC-3’	Sigma-Aldrich	Eps8L2_S181
5’-GAAGGCGCCAGCAGAGTAGGACCCAGCTTTCTTGTACAAAGTGGTCCCC-3’	Sigma-Aldrich	Eps8L2_R299
5’-GGACGCCCTCTGCTGGGGAGCCTGCTTTTTTGTACAAACTTGTCCCC-3’	Sigma-Aldrich	Eps8L2_S297
5’-CTCCGTCTCCTGCCCATAGGACCCAGCTTTCTTGTACAAAGTGGTCCCC-3’	Sigma-Aldrich	Eps8L2_R370
AGCAGTGGGCAGGAGACGGAGCCTGCTTTTTTGTACAAACTTGTCCCC-3’	Sigma-Aldrich	Eps8L2_S367
5’-ACCAGCCATGGCCAAATATTAGGACCCAGCTTTCTTGTACAAAGTGGTCCCC-3’	Sigma-Aldrich	Eps8L2_R496
5’-ACATATTTGGCCATGGCTGGGGAGCCTGCTTTTTTGTACAAACTTGTCCCC-3’	Sigma-Aldrich	Eps8L2_S494
5’-GTGCCCTGCAACATCCTATAGGACCCAGCTTTCTTGTACAAAGTGGTCCCC-3’	Sigma-Aldrich	Eps8L2_R546
5’-CGCCTCGCCTAGGATGTTGGAGCCTGCTTTTTTGTACAAACTTGTCCCC-3’	Sigma-Aldrich	Eps8L2_S544
5’-AGGGGGGAGGACAGCTAGGACCCAGCTTTCTTGTACAAAGTGGTCCCC-3’	Sigma-Aldrich	Eps8L2_R715
**Oligos used for RT-qPCRs**		
5’-AGGTGAAGAGGAAGATCCTG-3’	Sigma-Aldrich	Ctdnep1_Ex3_Ms_For
5’-TTGAGGATGAAGTCGGGAGG-3’	Sigma-Aldrich	Ctdnep1_Ex3_Ms_Rev
5´-GCACCTGGCCACATTCATCA-3’	Sigma-Aldrich	Eps8L2_Ex5_Ms_For
5’-ACTCAACATCCAGCAGTCGC-3’	Sigma-Aldrich	Eps8L2_Ex5_Ms_Rev
5’-AACTTTGGCATTGTGGAAGG-3’	Sigma-Aldrich	Gapdh_Ms_For
5’-ACACATTGGGGGTAGGAACA-3’	Sigma-Aldrich	Gapdh_Ms_Rev
**siRNAs**		
5’-GAUUCACUCUCACCACGAUTT-3’	ThermoFisher (Ambion)	Ctdnep1 siRNA #1

5’-GGUGGUAAUAGACAAACACTT-3’	Genecust	Ctdnep1 siRNA #2
5’-GCAGGUGAACGACAAGUCATT-3’	ThermoFisher (Ambion)	Eps8L2 siRNA #1
5’-GGAACAAAGAAGAGCUAAUTT-3’	ThermoFisher (Ambion)	Eps8L2 siRNA #2
5’-GAGAGUUUGUUGACUGUUUTT-3’	ThermoFisher (Ambion)	Eps8L2 siRNA #3
5’-CCAUCAUCCUGCACUUUCATT-3’	Genecust	Nesprin2G siRNA

Table S1. Oligonucleotides. Related to STAR Methods.
